# Essential role of autophagy in protecting neonatal haematopoietic stem cells from oxidative stress in a p62-independent manner

**DOI:** 10.1038/s41598-021-81076-z

**Published:** 2021-01-18

**Authors:** Naho Nomura, Chiaki Ito, Takako Ooshio, Yuko Tadokoro, Susumu Kohno, Masaya Ueno, Masahiko Kobayashi, Atsuko Kasahara, Yusuke Takase, Kenta Kurayoshi, Sha Si, Chiaki Takahashi, Masaaki Komatsu, Toru Yanagawa, Atsushi Hirao

**Affiliations:** 1 Division of Molecular Genetics, Cancer and Stem Cell Research Program, Cancer Research Institute, Kanazawa University, Kakuma-machi, Kanazawa, Ishikawa 920-1192 Japan; 2 WPI Nano Life Science Institute (WPI-Nano LSI), Kanazawa University, Kakuma-machi, Kanazawa, Ishikawa 920-1192 Japan; 3 Division of Oncology and Molecular Biology, Cancer and Stem Cell Research Program, Cancer Research Institute, Kanazawa University, Kakuma-machi, Kanazawa, Ishikawa 920-1192 Japan; 4 Institute for Frontier Science Initiative, Kanazawa University, Kakuma-machi, Kanazawa, Ishikawa 920-1192 Japan; 5 Department of Physiology, Juntendo University Graduate School of Medicine, 2-1-1, Hongo, Bunkyo-ku, Tokyo 113-8421 Japan; 6 Faculty of Medicine, University of Tsukuba, 1-1-1 Tennodai, Tsukuba, Ibaraki 305-8575 Japan; 7 Present Address: Department of Hematology, Chugoku Central Hospital, 148-13 Miyuki-cho, Kamiiwanari, Fukuyama, Hiroshima 720-0001 Japan; 8 Present Address: Division of Biomedical Oncology, Institute for Genetic Medicine, Hokkaido University, Kita-15, Nishi-7, Kita-ku Sapporo, Hokkaido 060-0815 Japan; 9 Present Address: Department of Pediatrics, Nagasaki Harbor Medical Center, 6-39 Shinchi-machi, Nagasaki City, Nagasaki 850-8555 Japan

**Keywords:** Haematopoiesis, Haematopoietic stem cells, Autophagy

## Abstract

Autophagy is a cellular degradation system contributing to homeostasis of tissue stem cells including haematopoietic stem cells (HSCs). It plays pleiotropic roles in HSC characteristics throughout life, but its stage-specific roles in HSC self-renewal are unclear. To investigate the effects of *Atg5* deletion on stage-specific HSC functions, we compared the repopulating capacity of HSCs in *Atg5*^*f*/*f*^;*Vavi-cre* mice from postnatal day (P) 0–7 weeks of age*.* Interestingly, Atg5 deficiency led to no remarkable abnormality in the HSC self-renewal capacity at P0, but significant defects at P7, followed by severe defects. Induction of *Atg5* deletion at P5 by tamoxifen administration to *Atg5*^*f*/*f*^;*Rosa26-Cre-ER*^*T2*^ mice resulted in normal haematopoiesis, including the HSC population, until around 1 year, suggesting that Atg5 in the early neonatal period was critical for haematopoiesis in adults. Mitochondrial oxidative stress was increased by Atg5 loss in neonatal HSC/progenitor cells. Although p62 had accumulated in immature bone marrow cells of *Atg5*^*f*/*f*^;*Vavi-cre* mice, *p62* deletion did not restore defective HSC functions, indicating that Atg5-dependent haematopoietic regulation in the developmental period was independent of p62. This study proposes a critical role of autophagy in HSC protection against harsh environments in the early neonatal stage, which is essential for healthy long-term haematopoiesis.

## Introduction

Autophagy is a conserved catabolic system that degrades cellular components engulfed in autophagosomes within lysosomes. This system is constitutively active to maintain cellular functions, but it is also induced in response to stress condition such as oxidative stress, infection, and starvation. Induced autophagy is a prosurvival mechanism to maintain cellular homeostasis by eliminating unwanted components such as damaged organelles, protein aggregates, and intracellular bacteria, and supplying an energy source. Autophagy consists of serial steps and different autophagy-related genes (Atgs) are involved in the process. It starts with emersion and expansion of an isolation membrane/phagophore. The phagophore encloses cytoplasmic components to form double membrane vesicles called autophagosomes. The autophagosomes then fuse with lysosomes to become autolysosomes in which degradation of cargo by lysosomal enzymes occurs. In mammals, for the initial step, the Unc-51 like autophagy activating kinase (ULK) complex consisting of ULK1 or ULK2, Atg13, FIP200, and Atg101 is recruited to sites near the endoplasmic reticulum (ER) and forms an assembly. Atg9-positive vesicles derived from the *trans*-Golgi network and plasma membrane localize to the ULK complex assembly and contribute to the formation of autophagosome precursors^1^. Phosphatidylinositol 3-kinase (PI3K) complex I consisting of Beclin1, Vps34, p150, Nrbf2, and Atg14L produces phosphatidylinositol 3-phosphate (PtdIns3P) in autophagosome-biogenesis-related membranes (*i.e.* autophagosome precursor, isolation membrane, and omegasome) to recruit multiple PtdIns3P-binding proteins that regulate autophagy formation^1^. The Atg12–Atg5–Atg16L1 complex, which is formed through a ubiquitin-like conjugation system involving Atg7 and Atg10, is recruited to the autophagosome precursor/isolation membrane via PtdIns3P-binding protein WIPI2b^1^. The Atg12–Atg5–Atg16L1 complex promotes conjugation of phosphatidylethanolamine to LC3 (Atg8 homolog in mammals), another ubiquitin-like conjugation system involving Atg4, Atg7, and Atg3^1,2^. The lipidation of LC3 has multiple roles in autophagy. For example, it is required for expansion of the isolation membrane and fusion of autophagosomes with lysosomes^1,3^. These two ubiquitin-like systems are crucial for successful autophagy.

Mammalian target of rapamycin (mTOR), a negative regulator of the autophagy pathway, which inactivates ULK1 by phosphorylation, is stimulated by insulin, growth factors, and amino acids. Lack of insulin or growth factors induces autophagy via the PI3K/Akt/mTOR axis, and a lack of amino acid induces it via regulation of Rag GTPase^4^, a component of mTOR complex 1 (mTORC1). mTOR also senses glucose starvation via the AMPK/mTOR axis to induce autophagy^4^. Generated amino acids are reused for protein and fatty acid biosynthesis, gluconeogenesis, and ATP production. Under infected conditions, attachment of bacteria to host cells^5^, bacterial pathogen-associated molecular patterns (PAMPs; e.g. lipopolysaccharide), and damage-associated molecular patterns (DAMPs; e.g. disturbed endosomal membrane)^6^, and bacterial nucleic acids (e.g. double-stranded DNA)^7^ trigger recruitment of autophagy machinery and rapid elimination of intracellular bacteria. Dysregulated autophagy is associated with pathogenesis in several tissues, typically neurons. In Alzheimer’s disease (AD), accumulation of amyloid beta peptide (Aβ) is thought to primarily trigger the pathogenesis. Growing evidence suggests that autophagy is a major pathway for degradation of Aβ, and dysfunction of autophagy contributes to the pathogenesis of AD^8^. Thus, in many situations, autophagy has important roles to protect cells from stress.

Although autophagy had been originally identified as a bulk degradation pathway, selective autophagic degradation has been identified. p62, also known as SQSTM1 (hereafter referred to as p62), is a major autophagic receptor that interacts with ubiquitinated substrates via the ubiquitin association domain and autophagosomes via the LC3 interaction region to intermediate selective engulfment of specific cargo into autophagosomes. p62 functions not only as an autophagic receptor, but also in the stress response. p62 activates nuclear factor erythroid 2-related factor (Nrf2) by competitive binding to Kelch-like ECH-associated protein (Keap) 1, promoting tumourigenesis in the liver^9^. Phosphorylation of S351 in p62 is observed in autophagic cargo, which increases affinity of p62 for Keap1. These findings suggest that selective autophagy and the Keap1-Nrf2 pathway are interdependent via p62^9^. In addition to mediating selective degradation of cargo, p62 itself is degraded by autophagy. Therefore, p62 accumulates under autophagy inhibitory conditions and in autophagy-deficient tissues. p62 accumulates in *Atg7*-deficient hepatocytes, and *p62* ablation suppresses liver injury and tumour development caused by autophagy deficiency^10–12^. However, accumulated p62 is not related to defective autophagy-deficient neurons^10^. The relationship of the phenotype caused by autophagy deficiency and p62 accumulation remains unclear.

Atg3, Atg5, Atg7, Atg12, and Atg16L1 are involved in the Atg12 conjugation system, and mice deficient for these genes (*Atg3*^−⁄−^, *Atg5*^−⁄−^, *Atg7*^−⁄−^, *Atg12*^−⁄−^, and *Atg16l1*^−⁄−^) are neonatal lethal and die within 1 day after birth^13–17^. *Atg5*-null mice (*Atg5*^*−⁄−*^), in which Atg5 is deficient in the whole body, fail to suckle milk and die within 1 day after birth^18^. Introduction of exogenous Atg5 to neurons in *Atg5*-null mice (*Atg5*^*−⁄−*^;*Eno2-Atg5*) recovers the suckling defect and neonatal lethality, suggesting that neuronal dysfunction triggers severe defects in *Atg5*-null mice. However, *Atg5*^*−⁄−*^;*Eno2-Atg5* mice, in which *Atg5* gene was restored only in neuron, start to die at around 2 months with abnormalities in multiple organs, indicating that autophagic activity is essential for development of tissues other than neuronal tissues. Additionally, because the amino acid level in *Atg5*-null neonates is lower and they died earlier than non-milk-fed wildtype neonates, metabolic and suckling defects trigger early neonatal lethality. Thus, it is still unclear how autophagy affects developmental processes.

To identify the role of autophagy in blood cells, various types of conditional *Atg* genes knockout mice have been used because of lethality of conventional *Atg* genes knockout mice^13–17^. It has been demonstrated that autophagy plays critical roles in the development and differentiation of various types of blood cells, including T, B, and erythroid cells^19–28^. Haematopoietic cell-specific *Atg5* and *Atg7* deletion in the foetal period using the Vav-Cre-loxP system triggers weight loss, severe anaemia, and reduced haematopoietic stem cells (HSCs) of mice and results in death^27–31^. In these mice, a mitochondrial status alteration has been observed^30,31^. In adult HSCs, for example, autophagy is induced rapidly upon starvation via the FoxO3a-driven gene expression program, which maintains the functionality of HSCs^32^. Furthermore, it has been suggested that autophagy degrades active healthy mitochondria and maintains low metabolic state and quiescence of HSCs^33^. However, knockout mice in which *Atg5* and *Atg12* genes re-deleted at juvenile to adult ages using the Mx1-Cre-loxP system are largely healthy, but show some phenotypes resembling aged mice, such as increased cellularity and a skewed ratio of myeloid versus lymphoid cells in peripheral blood (PB), indicating critical roles of autophagy in blood aging^33^.

In this study, we investigated the role of autophagy in HSC functions during developmental stages after birth using Atg5-deficient mice. Previous reports of *Atg5*^*f*/*f*^;*Vav-Cre* (*Atg5*^*f*/*f*^;*Vav*) or *Atg7*^*f*/*f*^;*Vav-Cre* mice have shown progressive defective haematopoiesis during developmental stages (6–9 weeks). Because the abnormalities of HSCs appeared to be more severe as the mice grew, it was assumed that autophagy is important for development of HSCs in later stages. However, the role of autophagy at early time points, such as the neonatal period, has not been clarified. In this study, by comparison of the regeneration capacity of HSCs among different timings after birth, we evaluated stage-specific effects of Atg5 deficiency on HSC functions. Additionally, because accumulation of p62 is observed in autophagy-deficient tissues^10–12^, we investigated the role of p62 in the response to oxidative stress induced by Atg5 deficiency in haematopoiesis by analysis of *Atg5*^*f*/*f*^;*p62*^*f*/*f*^;*Vav-Cre* (*Atg5*^*f*/*f*^;*p62*^*f*/*f*^;*Vav*) mice. As a result, we demonstrated that autophagy plays a critical role in protecting neonatal HSCs from oxidative stress in a p62-independent manner.

## Results

### Progressive haematopoietic failure by *Atg5* deletion during developmental period

**Figure 1:**
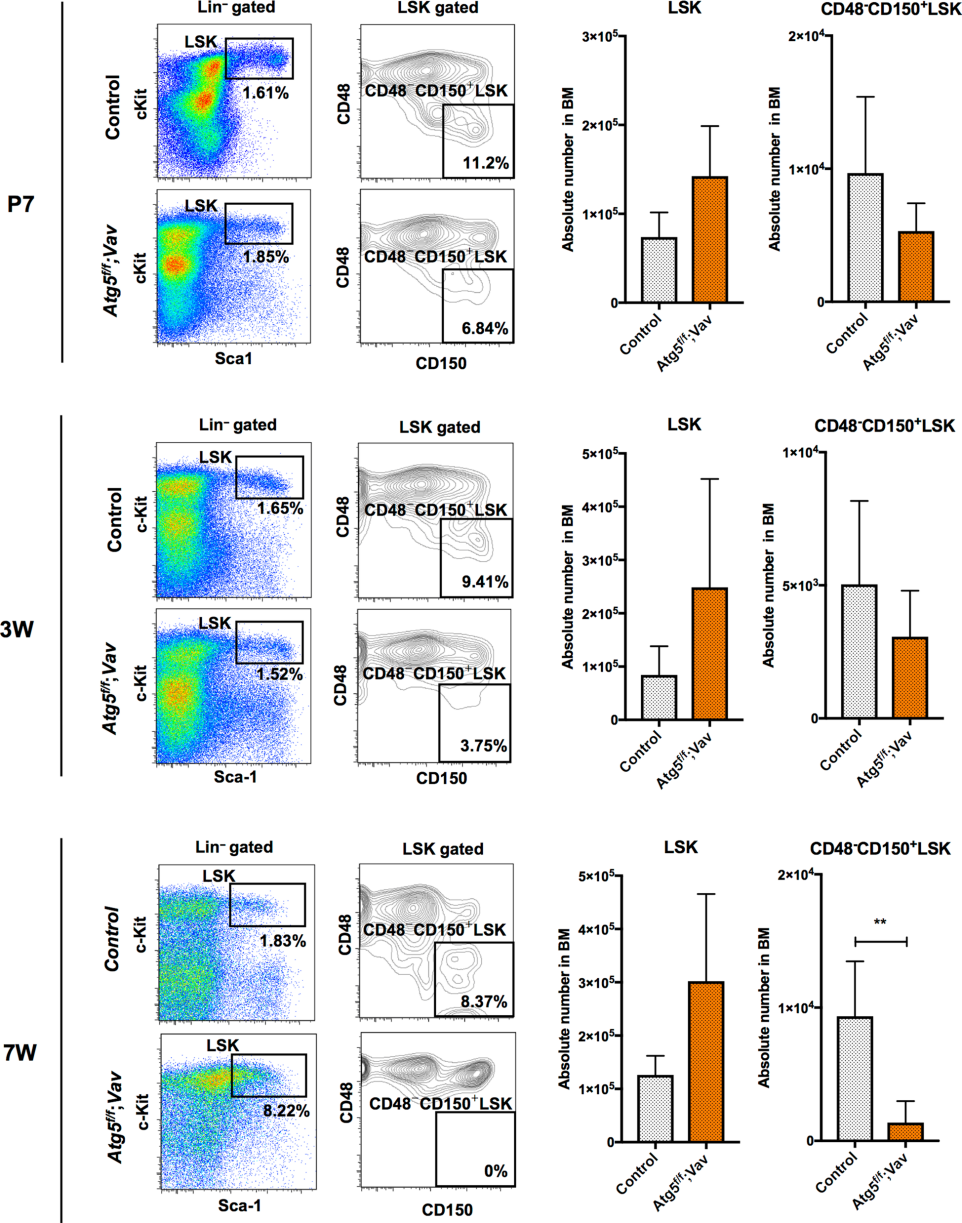
HSC population in control (*Atg5*^*f*/*w*^;*Vav*) and *Atg5*^*f*/*f*^;*Vav* mice at P7 (top), 3 weeks (middle), and 7 weeks (bottom). Left panel shows a representative flow cytometric plot of LSK (Lin^−^c-Kit^+^Sca1^+^) cells and CD48^−^CD150^+^LSK cells in at least three independent experiments. Right panel shows absolute numbers of LSK and CD48^−^CD150^+^LSK cells. Data are the mean ± SD (n = 3–6).

To investigate the roles of autophagy in development of haematopoiesis in mice, we analysed the effects of *Atg5* deletion on haematopoiesis using the Vav-Cre-loxP system, in which Cre recombinase-mediated loxP-flanked gene deletion in haematopoietic tissue starts from around embryonic day (E) 11.5^34^. The *Atg5* deletion efficiency was sufficiently high using this system (Supplementary Figure S1). Consistent with previous reports^29,30^, *Atg5*^*f⁄f*^;*Vav* mice showed severe haematopoietic failure at 7 weeks of age, and most Atg5-deficient mice died presumably because of further progression of haematopoietic failure (Supplementary Fig. S2). To evaluate the effect of Atg5 on HSC development, we investigated the long-term HSC population (SLAM LSK; CD48^−^CD150^+^lineage^−^Sca-1^+^c-Kit^+^) in P7, 3-week-old and 7-week-old mice. In bone marrow (BM) of 7-week-old mice, SLAM LSK cells were reduced dramatically by loss of the *Atg5* gene (Fig. 1) as shown previously^29,30^. This abnormality was not remarkable at earlier stages (Fig. 1). Therefore, as suggested in the previous reports, these data indicate that autophagy may play more critical roles in the later period rather than earlier developmental stages, such as the neonatal period.

### Defect in the self-renewal capacity of HSCs among BM cells of ***Atg5***^*f*/*f*^;***Vav*** mice at P7, but not P0

**Figure 2:**
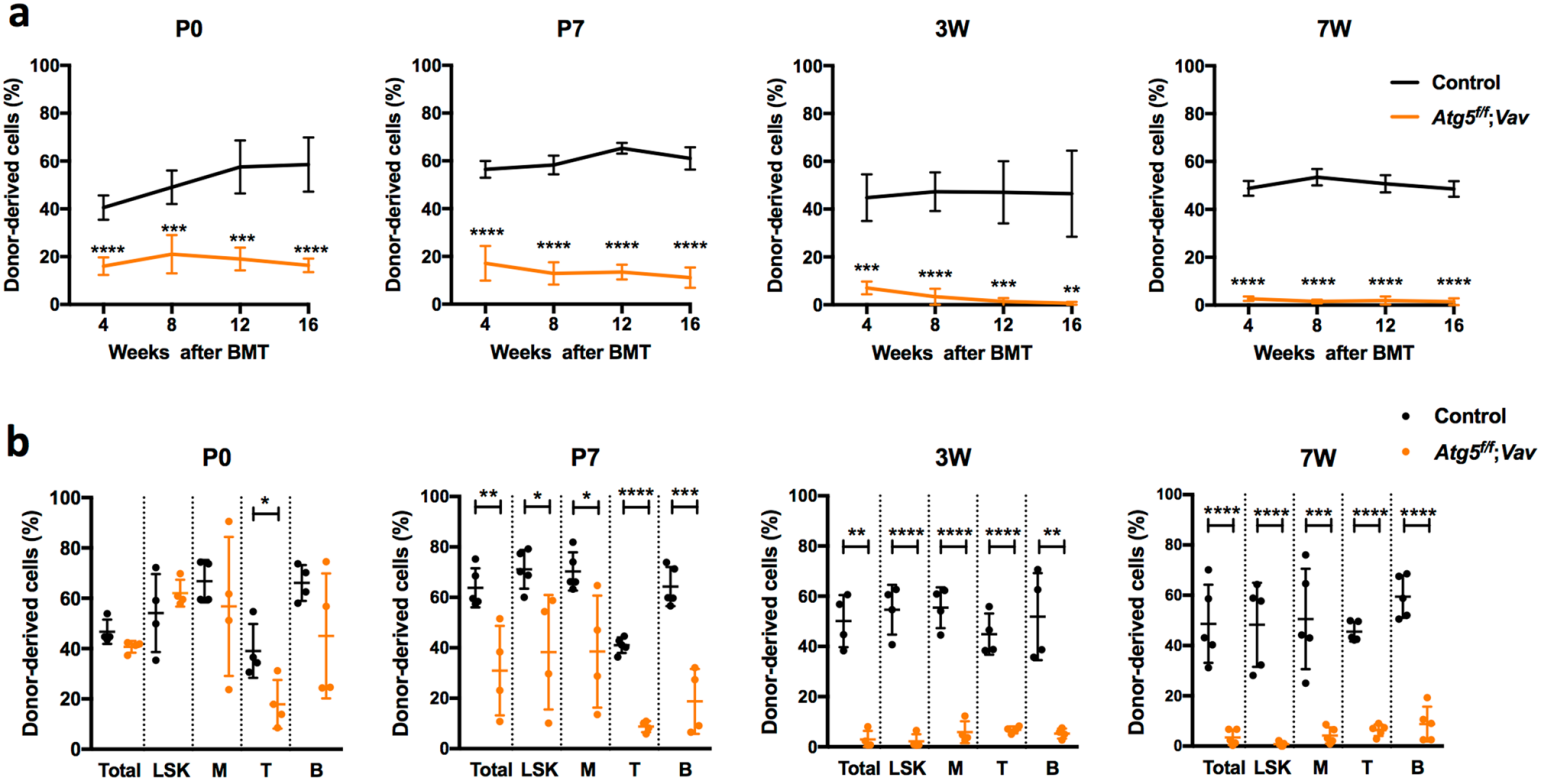
Competitive reconstitution analysis of BM cells from control (*Atg5*^*f*/*w*^;*Vav)* and *Atg5*^*f*/*f*^;*Vav* mice at P0, P7, 3 weeks, and 7 weeks. (**a**) Frequency of donor-derived cells among total PB cells were analysed every 4–16 weeks after transplantation. Data are the mean ± SD (n = 4–5). (**b**) Frequency of donor-derived cells among total, LSK, myeloid (M), T (T), and B (B) cells in BM were analysed at 16 weeks after transplantation. Data are the mean ± SD (n = 4–5). Horizontal line indicates the mean of values.

To evaluate the effect of Atg5 deficiency on HSC functions, we performed competitive reconstitution assays using BM cells of *Atg5*^*f*/*f*^;*Vav* mice from P0 to 7 weeks of age. When we transplanted BM from P0 to 7-week-old mice, all lineages derived from Atg5-deficient mice were reduced significantly in PB, which reduced further with age (Fig. 2a, Supplementary Fig. S3). In BM, regeneration of Atg5-deficient BM cells at 16 weeks after transplantation was reduced dramatically in 3- and 7-week-old BM cell recipients, indicating that Atg5-deficient mice lost their self-renewal capacity at 3 weeks of age (Fig. 2b). However, interestingly, its repopulation in BM was almost comparable to the control, when we transplanted Atg5-deficient BM cells at P0 (Fig. 2b). These data suggest that although production of mature cells in PB may be impaired by Atg5, development of immature cells was unaffected at P0. Hence, we analysed donor-derived HSC/progenitor cells (Lineage^−^Sca1^+^c-Kit^+^; LSK cells) at 16 weeks after transplantation to assess repopulation of HSCs, that is, their self-renewal capacity. We found that the frequency of donor-derived LSK cells was comparable to the control in transplantation of Atg5-deficient BM cells at P0, indicating that the self-renewal capacity of Atg5-deficient cells was normal at P0 (Fig. 2b). In transplantation of BM cells from P7 mice, we found a mild but significant reduction in repopulation of Atg5-deficient LSK cells, as well as myeloid, B, and T cells, indicating a reduction in the self-renewal capacity of HSCs (Fig. 2b). Thus, HSC dysfunction due to Atg5 loss was detected at P7, but not P0, followed by progressive dysfunction of HSCs in later stages.

### No remarkable abnormality in long-term haematopoiesis, including HSC functions, by *Atg5* deletion after P5

**Figure 3:**
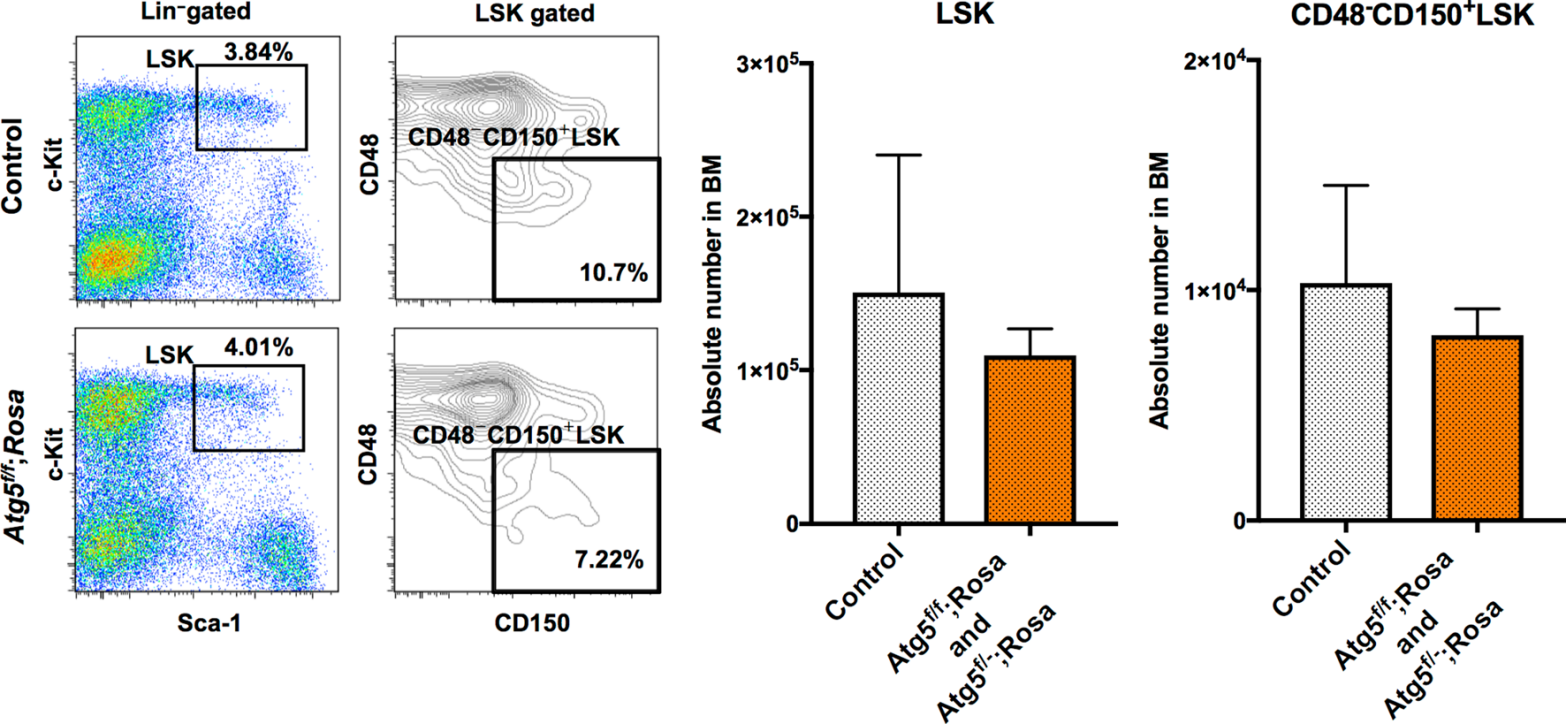
HSC population in control (*Atg5*^*f*/*w*^;*Rosa* and *Atg5*^*w*/*w*^;*Rosa*), and *Atg5*^*f*/*f*^;*Rosa* and *Atg5*^*f*/*−*^;*Rosa* mice at 8–12 months after tamoxifen administration at P5. Left panel shows a representative flow cytometric plot of LSK (Lin^−^c-Kit^+^Sca1^+^) cells and CD48^−^CD150^+^LSK cells in three independent experiments. Right panel shows absolute numbers of LSK and CD48^−^CD150^+^LSK cells. Data are the mean ± SD (n = 3).

Next, we investigated the effect of Atg5 loss on haematopoiesis, when it was induced at an earlier time after birth. For this purpose, we used two systems, *Atg5*^*f*/*f*^;*Mx1-Cre* (*Atg5*^*f*/*f*^;*Mx1*), and *Atg5*^*f*/*f*^;*Rosa26-Cre-ER*^*T2*^ (*Atg5*^*f*/*f*^;*Rosa*) or *Atg5*^*f*/*−*^;*Rosa26-Cre-ER*^*T2*^ (*Atg5*^*f*/*−*^;*Rosa*), in which *Atg5* gene deletion is induced by poly(I:C) and tamoxifen treatment, respectively. As the standard method, we started administration of poly(I:C) at 4 weeks of age and analysed haematopoiesis in adults. In our experiments, the Atg5 deficiency caused a mild but insignificant increase of LSK cells and LT-HSCs associated with increased myeloid cells and decreased B cells in PB, which is consistent with a previous report (Supplementary Fig. S4)^33^. These data indicated that deletion of *Atg5* gene in later developmental periods did not remarkably affect the HSC population. To delete the *Atg5* gene at the earliest timing after birth, we attempted to administrate tamoxifen at P5, followed by long-term observation of haematopoiesis. Although some mouse neonates died because of injury due to the intraperitoneal injection, most survived and appeared healthy. Analysis of PB showed normal haematopoiesis of Atg5-deficient mice. In some mice, we analysed HSCs and progenitors in BM after long-term observation (e.g., 8–12 months of age). The HSC and progenitor populations were comparable to the control (Fig. 3). To check the efficiency of gene deletion, we performed colony formation assays, followed by genotyping of individual colonies. We found that about 70% of colonies on average showed deletion of both alleles, indicating that the majority of LT-HSCs were deficient for Atg5 (Supplementary Fig. S5). Although there was the possibility that partial deletion of the *Atg5* gene may fail to result in remarkable phenotypes, these data support that Atg5 was dispensable to develop and maintain HSCs when the *Atg5* gene was deleted at around 1 week after birth.

### Increased mitochondrial oxidative stress by *Atg5* deletion is associated with cell death at the neonatal stage

**Figure 4:**
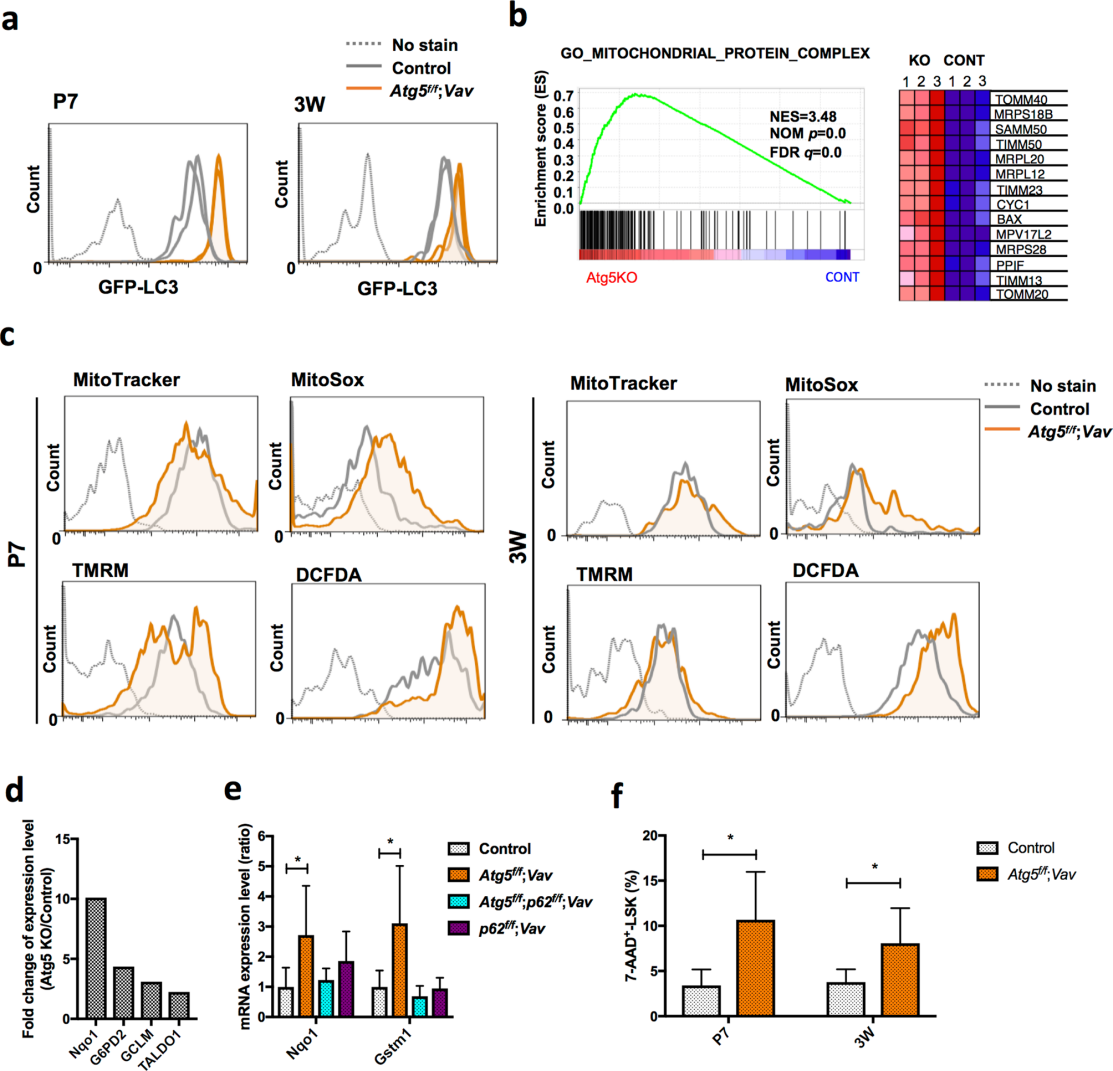
Gene expression and mitochondrial status in LSK cells from control and *Atg5*^*f*/*f*^;*Vav* mice. (**a**) Flow cytometric analysis of GFP-LC3 in LSK cells from control (*GFP-LC3*;*Atg5*^*f*/*w*^;*Vav* and *GFP-LC3*;*Atg5*^*f*/*w*^) and *GFP-LC3*;*Atg5*^*f*/*f*^;*Vav* mice at P7 and 3W. Representative data (two control and two *Atg5*^*f*/*f*^;*Vav*) from two independent experiments are shown (P7, n = 5–7; 3W, n = 3–5). (**b**) GSEA enrichment score curve for control (*Atg5*^*w*/*w*^;*Vav*) and *Atg5*^*f*/*f*^;*Vav* LSK cells analysed for the gene set associated with the mitochondrial protein complex. Heatmap of representative gene expression in the gene set is shown at the right. NES, normalised enrichment score; FDR, false discovery rate. (**c**) Flow cytometric analysis of MitoTracker green, MitoSox Red, TMRM, and DCFDA staining in LSK cells from control and *Atg5*^*f*/*f*^;*Vav* mice at P7 and 3 weeks. Representative data from at least three independent experiments are shown (n = 3–5). (**d**) Fold change of representative Nrf2-target gene expression levels upregulated in *Atg5*^*f*/*f*^;*Vav* LSK cells compared with control LSK cells from normalised microarray data. (**e**) mRNA expression levels of Nqo1 and Gstm1 in LSK cells from control, *Atg5*^*f*/*f*^;*Vav, p62*^*f*/*f*^;*Vav*, and *Atg5*^*f*/*f*^;*p62*^*f*/*f*^;*Vav* mice at 7 weeks. Data are the mean ± SD. n = 3–6. (**f**) Frequency of dead cells (7-AAD-positive cells) in LSK population from control (*Atg5*^*f*/*w*^;*Vav*) and *Atg5*^*f*/*f*^;*Vav* mice at P7 and 3W. Data are the mean ± SD (n = 4–5).

The above data prompted us to focus our analysis on HSCs at P7. First, to monitor autophagic activity in *Atg5*^*f*/*f*^;*Vav* and Control (*Atg5*^*f*/*w*^;*Vav* and *Atg5*^*f*/*w*^) mice, we used the GFP-LC3 system with FACS analysis^35,36^. If autophagy occurs, the GFP intensity decreases because it is digested and/or attenuated by the acidic condition in lysosomes in addition to LC3 protein, whereas GFP-LC3 accumulates upon autophagy inactivation. We compared the GFP fluorescence intensity in HSCs/progenitors (LSK) in *GFP-LC3*;*Atg5*^*f*/*f*^;*Vav* and *GFP-LC3*;*Atg5*^*f*/*w*^;*Vav* or *GFP-LC3*;*Atg5*^*f*/*w*^ mice. We found that *Atg5* deletion caused a clear increase of GFP intensity, which indicated that autophagy was impaired by *Atg5* deletion (Fig. 4a). Although, haematopoietic populations of Atg5-deficient mice appeared to be normal as shown in Fig. 1, we performed gene expression profiling to investigate mechanisms underlying damage of Atg5-deficient HSCs at around P7 (P6–8). GO term analysis using microarray data showed that the expression of genes related to mitochondria, such as *Tomm40* and *Mrps18b*, was significantly enriched in LSK cells from *Atg5*^*f⁄f*^;*Vav* mice (Fig. 4b, Supplementary Fig. S6). Additionally, we found elevated mitochondrial reactive oxygen species (ROS) detected by MitoSox staining in LSK cells of *Atg5*^*f⁄f*^;*Vav* mice at P7 (Fig. 4c). Increased mitochondrial ROS was also observed in LSK cells of 3-week-old *Atg5*^*f⁄f*^;*Vav* mice. We did not find an apparent increase of mitochondrial mass detected by MitoTracker staining or constant alteration of the mitochondrial membrane potential detected by TMRM staining caused by Atg5 deficiency at both P7 and 3 weeks of age. While p62 mediates selective degradation of autophagic cargo, it also has a role in the stress response through regulation of the Keap1-Nrf2 system that has been recognised as one of the major cellular defence mechanisms against oxidative and electrophilic stresses. It has been reported that p62 accumulation leads to activation of Nrf2, which induces many anti-oxidative genes^37^. It has also been reported that Nrf2 induces gene expression involved in anabolic pathways such as the pentose phosphate pathway^12,38^. As expected, we confirmed that p62 protein had accumulated in immature BM cells (lineage^−^c-Kit^+^) of *Atg5*^*f⁄f*^;*Vav* mice (Supplementary Fig. S7). Consistently, we found that Nrf2 target molecules, including *G6pd2* and *Taldo1* at around P7 (Fig. 4d) and *NAD(P)H dehydrogenase quinone 1* (*Nqo1*) and *glutathione S-transferase mu1* (*Gstm1*) at 7 weeks of age (Fig. 4e), were upregulated in LSK cells of Atg5-deficient mice. Furthermore, dead cells (7-AAD^+^) were increased by Atg5 (Fig. 4f). These data suggested that mitochondrial oxidative stress caused HSC damage in the neonatal stage, leading to a severe abnormality of haematopoiesis in adults as shown in Supplementary Fig. S2.

### No restoration of haematopoietic abnormality in Atg5-deficient mice by *p62* deletion

**Figure 5:**
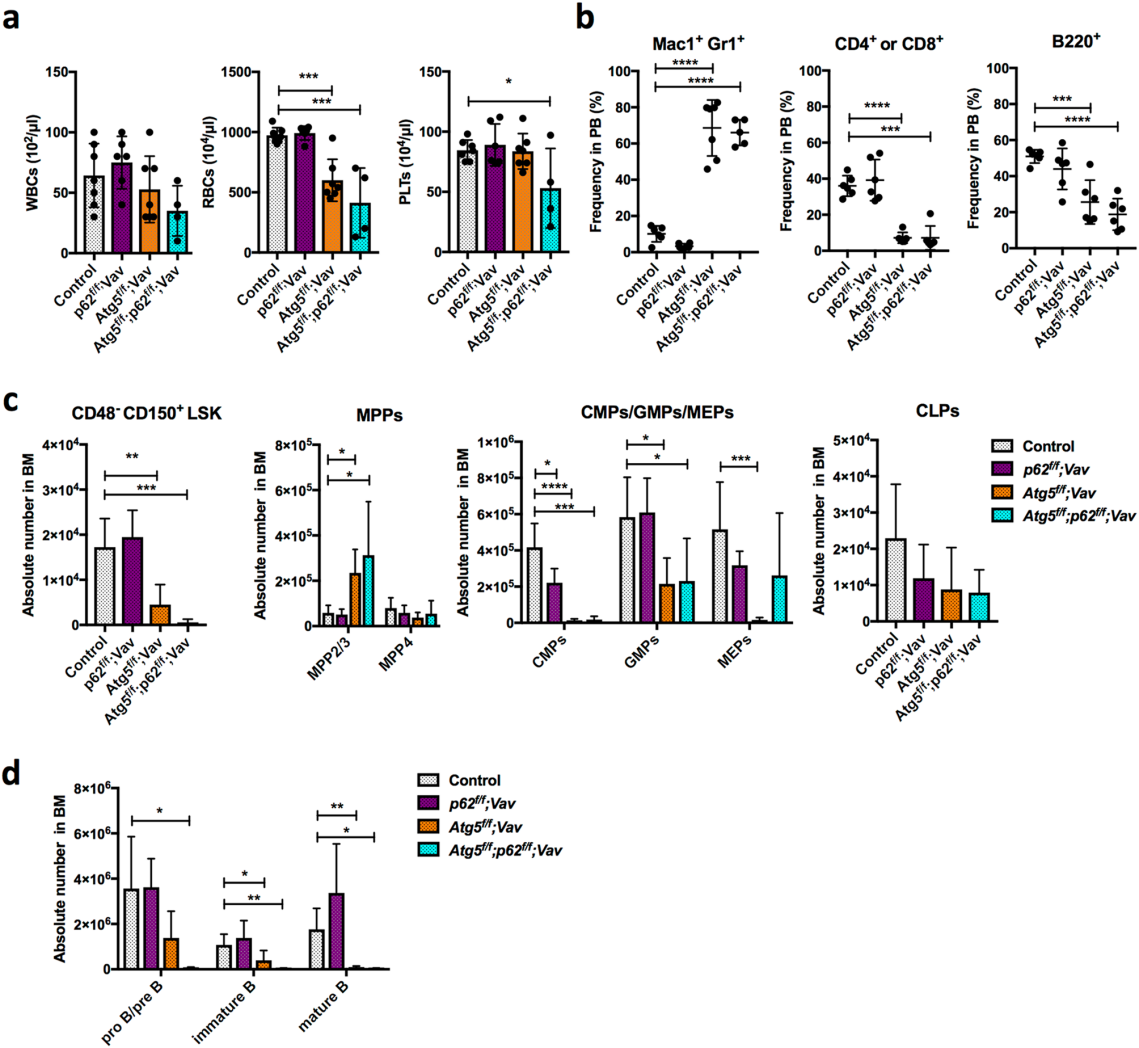
Comparative analysis of PB and BM cells from control, *p62*^*f*/*f*^;*Vav*, *Atg5*^*f*/*f*^;*Vav*, and *Atg5*^*f*/*f*^;*p62*^*f*/*f*^;*Vav* mice. (**a**) Counts of white blood cells (WBCs), red blood cells (RBCs), and platelets (PLTs) in PB. Data are the mean ± SD (n = 4–7). Each dot indicates values of individual mice. (**b**) Frequencies of myeloid cells (Mac1^+^Gr-1^+^), T cells (CD4^+^ or CD8^+^), and B cells (B220^+^) in PB. Data are the mean ± SD (n = 3–7). Horizontal line indicates the mean of values. (**c**) Absolute numbers of CD48^−^CD150^+^LSK cells, multipotent progenitors (MPPs; MPP2/3 and MPP4), common myeloid progenitors (CMPs), granulocyte macrophage progenitors (GMPs), megakaryocyte erythrocyte progenitors (MEPs), and common lymphoid progenitors (CLPs) in BM. Data are mean ± SD (n = 4–8). (**d**) Absolute numbers of mature B (CD43^−^B220^hi^IgM^+^), immature B (CD43^−^B220^lo^IgM^+^), and pro B/pre B (CD43^−^B220^lo^IgM^−^) cells in BM. Data are the mean ± SD (n = 3–6).

**Figure 6:**
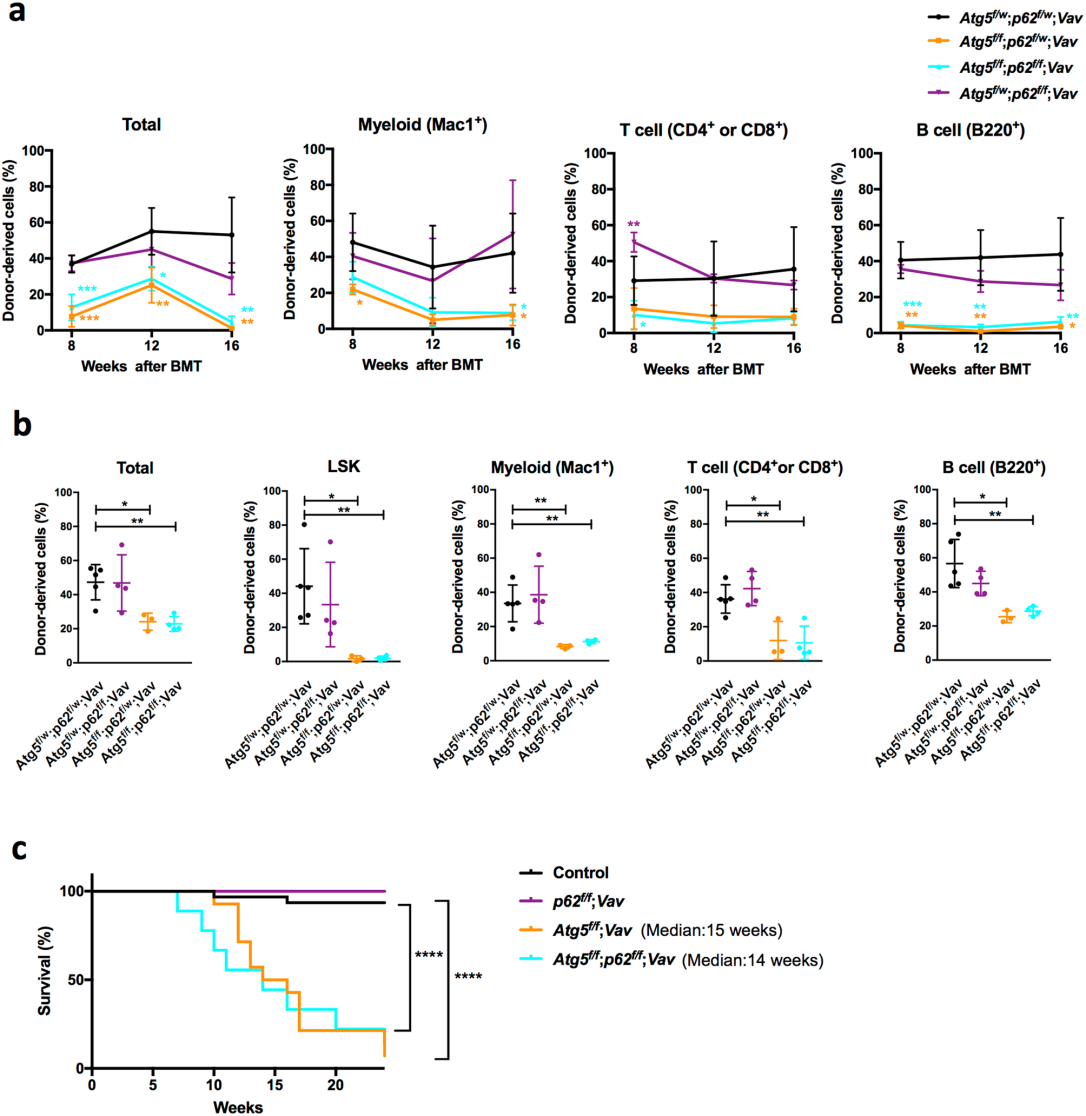
Competitive reconstitution analysis and survival assay. (**a**,**b**) Competitive reconstitution analysis of BM cells from *Atg5*^*f*/*w*^;*p62*^*f*/*w*^;*Vav*,*Atg5*^*f*/*f*^;*p62*^*f*/*w*^;*Vav*,*Atg5*^*f*/*f*^;*p62*^*f*/*f*^;*Vav*, and *Atg5*^*f*/*w*^;*p62*^*f*/*f*^;*Vav* mice at 3 weeks. (**a**) Frequencies of donor-derived cells among total cells and lineage cells in PB were analysed 8–16 weeks after transplantation. Data are the mean ± SD (n = 3–5). (**b**) Frequencies of donor-derived cells among total, LSK, myeloid, T, and B cells in BM were analysed 16 weeks after transplantation. Data are the mean ± SD (n = 3–5). Horizontal line indicates the mean of values. (**c**) Survival curve of control (n = 31), *p62*^*f*/*f*^; *Vav* (n = 6), *Atg5V*; *Vav* (n = 13), and *Atg5*^*f*/*f*^; *p62*^*f*/*f*^; *Vav* (n = 9) mice.

**Figure 7:**
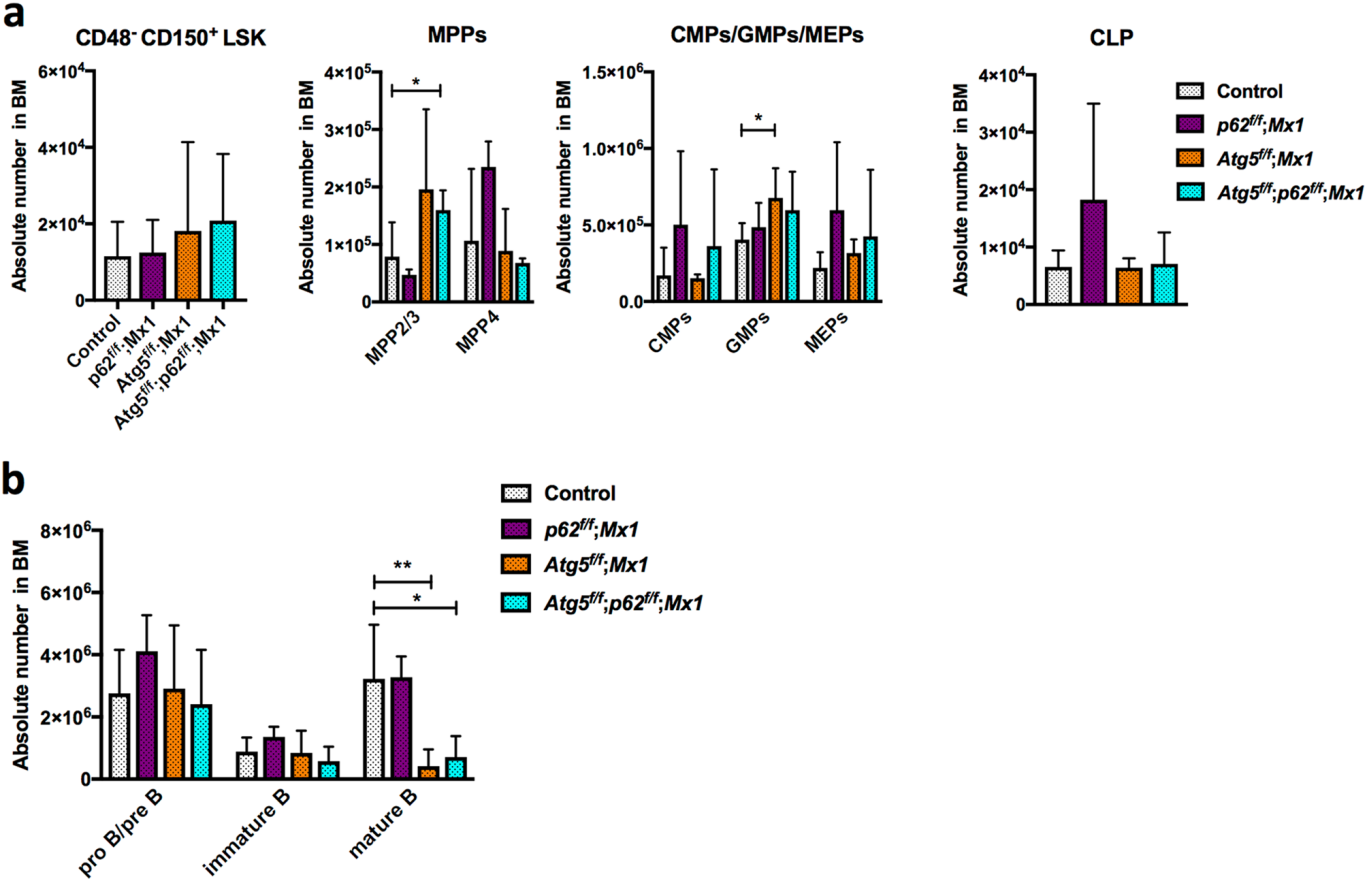
Comparative analysis of HSCs, progenitors, and B cells in BM from control, *p62*^*f*/*f*^; *Mx1*, *Atg5*^*f*/*f*^; *Mx1*, and *Atg5*^*f*/*f*^; *p62*^*f*/*f*^; *Mx1* mice. (**a**) Absolute numbers of CD48^−^CD150^+^LSK cells, multipotent progenitors (MPPs; MPP2/3 and MPP4), common myeloid progenitors (CMPs), granulocyte macrophage progenitors (GMPs), megakaryocyte erythrocyte progenitors (MEPs), and common lymphoid progenitors (CLPs) in BM. Data are the mean ± SD (n = 4–7). (**b**) Absolute numbers of mature B (CD43^−^B220^hi^IgM^+^), immature B (CD43^−^B220^lo^IgM^+^), and pro B/pre B (CD43^−^B220^lo^IgM^−^) cells in BM. Data are the mean ± SD (n = 5–7).

Finally, we investigated whether p62 was involved in the phenotype of *Atg5* deletion by the Vav-Cre-loxP system. We confirmed sufficiently high efficiency of p62 deletion using this system (Supplementary Figure S1). *p62*^*f*/*f*^;*Vav-Cre* (*p62*^*f*/*f*^;*Vav*) mice appeared healthy and did not show any haematopoietic abnormalities such as anaemia or an altered distribution of lineage cells in PB (Fig. 5a,b). In BM, HSC/progenitor populations were almost comparable to the control, except for mildly reduced CMP in *p62*^*f*/*f*^;*Vav* mice at 7 weeks of age (Fig. 5c,d). Myeloid cells and lymphocytes in BM and spleen were normal (Supplementary Fig. S8). Reconstitution assays showed that the functions of p62-deficient HSCs, including self-renewal, were not impaired (Supplementary Fig. S8). Thus, p62 loss alone barely affected haematopoiesis. Hence, we assessed the effects of loss of p62 on haematopoiesis in *Atg5*^*f⁄f*^;*Vav* mice at 7 weeks of age. As shown in Supplementary Fig. S2, *Atg5*^*f⁄f*^;*Vav* mice at 7 weeks of age showed severe haematopoietic dysfunctions such as severe anaemia and leukocytopenia associated with an abnormality in balance of peripheral cell components, such as increased ratios of myeloid/B cells, compared with control (*Atg5*^*w*/*w*^;*Vav*) mice (Fig. 5a,b). In *Atg5*^*f⁄f*^;*Vav* mouse BM, in addition to reduced LT-HSCs^29,30^, significant increases of myeloid-biased multipotent progenitor (MPP) 2 and MPP3 were observed (Fig. 5c). Consistent with previous reports, granulocyte–macrophage progenitors (GMPs) were reduced significantly^30^, and common myeloid progenitors (CMPs)/megakaryocyte-erythrocyte progenitors (MEPs) were reduced similarly. Additionally, we found that mature B cells in BM were reduced drastically in *Atg5*^*f⁄f*^;*Vav* mice (Fig. 5d). In *Atg5*^*f*/*f*^;*p62*^*f*/*f*^;*Vav* mice, most haematopoietic parameters in PB, BM, and spleen appeared to be comparable with *Atg5*^*f*/*f*^;*Vav* mice (Fig. 5a–c, Supplementary Fig. S8), indicating that loss of p62 did not restore the defective haematopoiesis due to Atg5 deficiency. Interestingly, we found that *Atg5*^*f*/*f*^;*p62*^*f*/*f*^;*Vav* mice consistently exhibited more severe phenotypes, particularly in the LT-HSC population as well as proB/preB and immature B cells, suggesting that Atg5 and p62 partly support HSC functions in a collaborative manner. To investigate the roles of p62 in defective phenotypes of Atg5 deficiency, we performed reconstitution assays using *Atg5*^*f⁄f*^;*p62*^*f⁄f*^;*Vav* mice. While the repopulating capacity in all lineage was reduced significantly by Atg5 loss both in PB and BM of recipients, p62 loss did not restore it (Fig. 6a,b). Because of severe haematopoietic defects, *Atg5*^*f⁄f*^;*Vav* mice survived for 15 weeks on average, and lethality of Atg5/p62-deficient mice was also comparable to that of Atg5-deficient mice (Fig. 6c). Thus, we concluded that Atg5-dependent haematopoietic regulation during the developmental period was independent of p62. Additionally, we evaluated the effects of *p62* deletion in Atg5-deficient mice that lost *Atg5* after birth by poly (I:C) administration using the Mx1-Cre-loxP system. Consistent with a previous report^33^, *Atg5*^*f*/*f*^;*Mx1* mice showed a slight increase of GMPs, but it was largely comparable with the control for the HSC/progenitor population (Fig. 7a). In lineage cells, a slight increase of splenic myeloid cells and altered erythroid development were observed (Supplementary Fig. S9). Apart from *Atg5*^*f*/*f*^;*Vav* mice, *Atg5*^*f*/*f*^;*Mx1* mice also showed a reduction of mature B cells (Fig. 7b). These alterations were not restored by *p62* deletion (Fig. 7a,b, Supplementary Fig. S9 and S10). In summary, haematopoietic regulation by Atg5 during both developmental and adult periods was independent of p62.

## Discussion

In mice, primitive haematopoiesis begins in blood islands of the yolk sac at E7, followed by the switch from primitive to definitive haematopoiesis at E10 and 11. At the beginning of the perinatal period, the liver is still a source of extra-medullary haematopoiesis, and then the BM eventually takes over^39^. During the perinatal period, foetal and neonatal development and maturation are deeply affected by environmental conditions such as oxidative stress, nutritional stress, and microbial infection. Although it has been demonstrated that autophagy plays a critical role in development and aging of HSCs, the importance of autophagy in perinatal HSCs, especially during the neonatal period, has not been revealed. Based on this study, we propose a critical role of autophagy in protection of HSCs against harsh environments in the early neonatal stage, which is essential for healthy long-term haematopoiesis. The perinatal period is accompanied by dramatic environmental changes in both oxidative stress and nutritional conditions, which may affect autophagic activity. Because the transplacental nutrient supply is suddenly interrupted at birth, neonates have stress due to starving until milk feeding. Foetal life evolves under a hypoxic condition, but it provides comfortable environments for development of organs. During foetal-to-neonatal transition, asphyxia is characterised by periods of severe hypoxia that may evolve to ischemic organ impairment. In another aspect of oxygen, foetal-to-neonatal transition may cause “relative hyperoxia”. In both cases, oxidative stress occurs in neonatal tissues. Although it is unclear which factors activate autophagy, it is assumed that autophagy is important for survival of HSCs against specific harsh neonatal environments. To understand the neonatal period better, we analyzed public RNA-seq data from wild type neonatal (P7) HSCs (CD48^-^CD150^+^Lineage^-^Sca-1^+^c-Kit^-^) and adult HSCs deposited in GEO (GSE128762)^40^. We found that expression of many genes involved in glucose and glutamine metabolisms, mitochondria, and redox regulation varied significantly between at P7 and adult mice. The expression levels of most of these genes are significantly lower in neonatal HSCs compared with those in adult HSCs (Supplementary Fig. S11). It has been reported that the metabolism and redox regulation are closely related to the regulation and maintenance of HSCs in adults^41–43^. Mitochondria, a dynamic organelle in which many metabolic processes occur, also have critical roles in maintenance of HSCs^44^. The lower expression of these genes in HSCs during neonatal period suggest that the protective system of HSCs against metabolic stress may be somewhat different among the developmental stages, and that survival of neonatal HSCs may be highly dependent on autophagy. Additional analysis is needed to understand the stage-specific roles of autophagy.

Further understanding of the roles of autophagy in neonates may uncover novel pathophysiological insights of disease that caused by autophagy-related abnormalities in the neonatal period. One such insight may be the mitochondrial behaviour of HSCs. Accumulating evidence has shown that HSCs with increased and active mitochondria have loss of haematopoietic functions such as the reconstitution ability^45–47^. In our study, alteration of the TMRM level was not apparent at P7 and 3 weeks of age, although an increased mitochondrial mass, TMRM level, and mitochondrial ROS were observed at 7 weeks (Supplementary Fig. S12) consistent with previous data^30^. Although we do not have clear evidence of the roles of oxidative stress in defective phenotypes of HSCs in *Atg5*^*f*/*f*^;*Vav* mice, we assume that the functional change of mitochondria may occur at the perinatal stage. Further analysis will be needed to understand the molecular mechanism.

In this study, we focused on the role of p62 in autophagy-dependent anti-oxidative effects on HSCs. p62 is a signalling hub that interacts with various partners. Previous functional analyses of p62 knockout (*p62*^*−⁄−*^) mice indicated that the main phenotype of the mice is obesity^48^. *p62*^*−⁄−*^ mice do not show haematopoietic failure for up to 6 months after birth or transplantation^49^. Consistently, our analysis of *p62*^*f*/*f*^;*Vav* mice showed that the haematopoietic stem cells were normal and their reconstitution ability were maintained (Fig. 5c and Supplementary Fig. S8). Because it has been reported that p62 is required for amino acid sensing via the mTOR pathway^50^, p62 might play a critical role in the protective response to abnormalities in amino acid levels. Additionally, we analysed Atg5/p62-deficient HSCs. *p62* ablation suppresses the hepatic phenotype, but not affect the neuronal defect caused by autophagy^10–12^. This study clarified that p62 was not the cause of the severe functional decline of VavCre-mediated Atg5-deficient HSCs. Although p62 accumulated and induced Nrf2-targeted gene expression in HSCs derived from *Atg5*^*f*/*f*^;*Vav* mice (Supplementary Fig. S7 and Fig. 4d,e), the gene induction was not remarkable compared with Atg5-deficient hepatic cells^12^. Because autophagy has pleiotropic roles in haematopoiesis, we also analysed other haematopoietic cells in addition to HSCs. For example, we found severe impairment of B cell development in *Atg5*^*f*/*f*^;*Vav* mice, which was consistent with the phenotype of B cell-specific Atg5-deficient mice^23^, but p62 loss did not rescue it. *Atg5*^*f*/*f*^;*Mx1* mice also showed impairment of B cell development, but the phenotype was much more milder than that of *Atg5*^*f*/*f*^;*Vav* mice. p62 loss did not rescue impaired B cell development in *Atg5*^*f*/*f*^;*Mx1* mice. In some cases, concomitant deletion of *p62* worsens the phenotype of Atg5 loss, suggesting that p62-mediated pathways collaborate with autophagy for proper HSC development. One possibility is that p62 may have a protective role mediating mTOR signalling under autophagy-deficient conditions. It has been reported that p62 interacts with mTORC1 components Raptor and Rags, and mediates amino acid sensing^50^. mTORC1 has an essential role in HSC functions in vivo^51,52^. Because p62 deficiency did not cause apparent haematopoietic abnormalities except for a mild reduction of CMP, autophagy and p62 may act synergistically for HSC functions, at least in part. Further analysis is needed to understand how HSCs normally develop in the harsh environment during the neonatal period.

In conclusion, we demonstrated that autophagy plays a critical role in protecting neonatal HSCs from oxidative stress in a p62-independent manner. Our findings provide novel insights regarding a critical role of autophagy in protection of HSCs against harsh environments in the early neonatal stage, which is essential for healthy long-term haematopoiesis in adults.

## Materials and methods

### Mice

*Atg5*^*flox*/*flox*^ (*Atg5*^*f*/*f*^) mice^53^ were bred with *Vavi-Cre*^54^ (Charles River Laboratories Japan Inc.,) or *Mx1-Cre*^55^ transgenic mice to obtain *Atg5*^*f*/*f*^;*Vavi-Cre* (*Atg5*^*f*/*f*^;*Vav*) and *Atg5*^*f*/*f*^;*Mx1-Cre* (*Atg5*^*f*/*f*^;*Mx1*) mice, respectively. *p62*^*flox*/*flox*^ (*p62*^*f*/*f*^)^56^ mice were bred with *Atg5*^*f*/*f*^;*Vav* and *Atg5*^*f*/*f*^;*Mx1* mice to obtain *Atg5*^*f*/*f*^;*p62*^*f*/*f*^*;Vav* and *Atg5*^*f*/*f*^;*p62*^*f*/*f*^;*Mx1* mice. *Atg5*^*f*/*f*^ mice were bred with *Rosa26-CreER*^*T2*^^57^ to obtain *Atg5*^*flox*/*flox*^;*Rosa26-CreER*^*T2*^ (*Atg5*^*f*/*f*^;*Rosa*) mice. *Atg5*^*flox*/*wild*^;*Rosa26-CreER*^*T2*^ (*Atg5*^*f*/*w*^;*Rosa*) mice were administrated tamoxifen to delete the floxed *Atg5* gene. *Atg5*^*−*/*w*^;*Rosa* mice were bred with *Atg5*^*f*/*w*^;*Rosa* or *Atg5*^*f*/*f*^;*Rosa* to obtain *Atg5*^*f*/*−*^;*Rosa* mice. *Atg5*^*f*/*f*^;*Vavi-Cre* mice were bred with CAG-GFP-LC3 mice (RBRC00806, provided by RIKEN BRC through the National BioResource Project of the MEXT/AMED, Japan) to obtain *GFP-LC3*;*Atg5*^*f*/*f*^;*Vavi-Cre* mice. Genotyping was performed on tail or PB genomic DNA. PCR primers were as follows:

*Atg5* WT/floxed/deleted alleles, 5′-ATGGTGTCTCCCACATCAGTTAGC-3′, 5′-CCAATACACAGGGTACACTGAAGG-3′, 5′-AGTGAAGGAGTGGAAAGTAGTGGG-3′; *p62* WT/floxed/deleted alleles, 5′-AGGCTGGCACAGTGAATCTT-3′, 5′-TCTGTGTCCTCCTAGCTTCT-3′, 5′-TCAATTCCCAGTACTGGAGG-3′; *Vav-iCre*, 5′-AGATGCCAGGACATCAGGAACCTG-3′, 5′-ATCAGCCACACCAGACACAGAGATC-3′; *Mx1-Cre*^58^, 5′-TCCCAACCTCAGTACCAAGC-3′, 5′-ATTCTCCCACCGTCAGTACG-3′; *Rosa26-Cre-ER*^*T2*^, 5′-AAAGTCGCTCTGAGTTGTTAT-3′, 5′-CCTGATCCTGGCAATTTCG-3′; *GFP-LC3*, 5′-ATAACTTGCTGGCCTTTCCACT-3′, 5′-CGGGCCATTTACCGTAAGTTAT-3′, 5′-GCAGCTCATTGCTGTTCCTCAA-3′.

For Mx1-Cre-mediated deletion, 4-week-old mice were injected intraperitoneally three times 2 days apart with 400 μg poly (I:C) (Sigma-Aldrich). Mx1-Cre mice were analysed at 4 weeks after poly (I:C) administration. For *Rosa26-Cre-ER*^*T2*^ mediated deletion, P5 neonates were injected intraperitoneally with 0.5 mg tamoxifen. The mice were analysed at 8–12 months after tamoxifen administration. To determine gene deletion efficiency, colony PCR was conducted. Sorted LSK cells were cultured in Methocult GF M3434 (STEMCELL Technologies) and single cell-derived colonies were picked up. Cells were suspended in 0.01 M Tris–HCl (pH 8.0) containing proteinase K (200 μg/ml) and incubated for 90 min at 56 °C and then for 10 min at 90 °C. DNA was obtained and analysed by PCR.

Male and female mice were used equally in all experiments. In experiments using *Atg5*^*f*/*f*^;*Vav* mice, *Atg5*^*f*/*w*^;*Vav*, *Atg5*^*w*/*w*^;*Vav* and *Vav*-negative littermates (*Atg5*^*f*/*f*^ and *Atg5*^*f*/*w*^) were used equally as controls unless otherwise specified in figure legends. In experiments using *Atg5*^*f*/*f*^;*p62*^*f*/*f*^;*Vav* mice, *Atg5*^*f*/*w*^;*p62*^*f*/*w*^;*Vav*, *Atg5*^*f*/*w*^;*p62*^*w*/*w*^;*Vav*, *Atg5*^*w*/*w*^;*p62*^*f*/*w*^;*Vav*, *Atg5*^*w*/*w*^;*p62*^*w*/*w*^;*Vav*, and *Vav-*negative littermates were used equally as controls unless otherwise specified in figure legends. In experiments using *Atg5*^*f*/*f*^;*Rosa* and *Atg5*^*f*/*−*^;*Rosa* mice, *Atg5*^*f*/*w*^;*Rosa* and *Atg5*^*w*/*w*^;*Rosa* littermates were used as controls. In experiments using *Atg5*^*f*/*f*^;*Mx1* mice, *Atg5*^*f*/*w*^;*Mx1* and *Atg5*^*f*/*f*^ littermates were used as controls unless otherwise specified in figure legends. In experiments using *Atg5*^*f*/*f*^;*p62*^*f*/*f*^;*Mx1* mice, *Atg5*^*f*/*w*^;*p62*^*w*/*w*^;*Mx1*, *Atg5*^*w*/*w*^;*p62*^*w*/*w*^;*Mx1*, and *Mx1*-negative littermates were used equally as controls. All animal experiments were approved by the Committee on Animal Experimentation of Kanazawa University, and performed in accordance with the Guidelines for Animal and Recombinant DNA Experiments of Kanazawa University.

### Haematological analysis

PB was collected from the postorbital vein and suspended in a heparin solution. Complete blood count analysis was performed using a Celltac alpha (Nihon Kohden). Before antibody staining and DNA extraction, erythrocytes were lysed with ammonium chloride. BM cells were obtained from femoral and tibial bones of hind legs by flushing the BM cavity. If necessary, isolation of mononuclear cells by density gradient centrifugation using Lymphoprep (Axis-Shield) was conducted. BM cells were stained with antibodies for flow cytometric analysis or used for transplantation.

### Flow cytometry

PB and BM cells were stained with monoclonal rat anti-mouse antibodies recognising each marker. Fc-block (BD Biosciences) was used to block non-specific binding of antibodies to Fcγ receptor. To stain HSCs, a mixture of biotinylated antibodies for lineage cells, streptavidin-APC780 (eBioscience), anti-c-Kit-APC (BD Biosciences), anti-Sca1-PECy7 (BD Biosciences), anti-CD48-FITC (BD Biosciences), and anti-CD150-PE (BioLegend) antibodies were used. To stain HSCs from mice at < 4 weeks of age, biotinylated anti-Gr-1 (eBioscience), anti-Ter119 (eBioscience), anti-IL7R (BD Biosciences), anti-B220 (eBioscience), anti-CD4 (eBioscience), and anti-CD8 (eBioscience) antibodies were used for lineage cell staining. To stain HSCs and progenitors (MPPs, CMP/GMP/MEPs, and CLPs) from mice at ≥ 4 weeks of age, the biotinylated anti-Mac-1 (eBioscience) antibody was added to the biotinylated antibody mixture for lineage cells. To stain MPPs, an anti-CD135-BV421 (BD Biosciences) antibody was combined with HSC-staining antibodies. For CMP/GMP/MEP staining, the mixture of biotinylated antibodies for lineage cells, streptavidin-APC780 (eBioscience), anti-c-Kit-APC (BD Biosciences), anti-Sca1-PECy7 (BD Biosciences), anti-FcγR-PE (BD Biosciences), and anti-CD34-FITC (BD Biosciences) antibodies were used. For CLP staining, the mixture of biotinylated antibodies for lineage cells except for IL7R, streptavidin-APC780 (eBioscience), anti-c-Kit-APC (BD Biosciences), anti-Sca1-PE (BD Biosciences), and anti-IL7R-PECy7 (eBioscience) antibodies were used. To analyse B cell development, biotinylated anti-CD43 (eBioscience), streptavidin-APC780 (eBioscience), anti-B220-FITC (BD Biosciences), and anti-IgM-APC (BD Biosciences) antibodies were used. For myeloid cell staining, anti-Mac1-FITC (BD Biosciences), anti-Gr-1-PE (eBioscience), or anti-Gr-1-PECy7 (eBioscience) antibodies were used. For lymphoid cell staining, anti-B220-FITC (BD Biosciences) or anti-B220-PECy7 (eBioscience), anti-CD4-APC (BD Biosciences), anti-CD8-APC (BD Biosciences), or anti-CD8-PE (BD Biosciences) antibodies were used. For erythroid cell staining, anti-Ter119-PE (BD Biosciences) and anti-CD71-FITC (eBioscience) antibodies were used. For LSK chimerism analysis, biotinylated antibodies for lineage cells, streptavidin-APC780 (eBioscience), anti-c-Kit-APC (BD Biosciences), anti-Sca-1PECy7 (BD Biosciences), anti-CD45.1-PE (BD Biosciences), and anti-CD45.2-FITC (BD Biosciences) antibodies were used. For myeloid chimerism analysis, anti-Mac-1APC (eBioscience), anti-Gr-1-PECy7 (eBioscience), anti-CD45.1-PE (BD Biosciences), anti-CD45.2-FITC (BD Biosciences), or anti-Mac1-PB (BioLegend), anti-Gr-1APC (BD Biosciences), anti-CD45.1-PE (BD Biosciences), and anti-CD45.2-FITC (BD Biosciences) antibodies were used. For lymphoid chimerism analysis, anti-B220-PECy7 (eBioscience) or anti-B220-PB (BD Biosciences), anti-CD4-APC (BD Biosciences), anti-CD8-APC (BD Biosciences), anti-CD45.2-FITC (BD Biosciences), and anti-CD45.1-PE (BD Biosciences) antibodies were used. Stained cells were washed with PBS containing 5% FCS, and dead cells were stained with 7-AAD (BD Biosciences). Samples were analysed using a FACSCanto II, FACSLyric, or FACSAria II/III (Becton Dickinson).

### Transplantation

For BM cell transplantation, donor (CD45.2) whole BM cells (1 × 10^6^/recipient) were mixed with competitor (CD45.1) whole BM cells (1 × 10^6^/recipient) and transplanted into lethally irradiated (9 Gy) recipient (CD45.1) mice through the tail vein. Donor-derived cells were recognised by CD45.2 expression.

### Western blotting

c-Kit^+^Lin^−^ cells (5 × 10^6^) were sorted by a FACSAria II and lysed with RIPA buffer (25 mM Tris–HCl, 150 mM NaCl, 1% sodium deoxycholate, 0.1% SDS, and 1% NP40) supplemented with Complete Mini (Roche). After mixing with SDS Sample buffer, proteins were denatured by boiling. Denatured proteins were separated on a 15% acrylamide gel and then transferred to a PVDF membrane. After blocking with 5% dry skim milk in PBST, blots were incubated with Abs against ATG5 (Novus Biologicals)^59^, p62 (Abnova)^60^, LC3 (Nanotools)^61^, and β-actin (Sigma-Aldrich)^62^. Immunocomplexes were then labelled with a HRP-conjugated anti-mouse, or with anti-rabbit antibodies, and visualised using ECL Prime Western Blotting Detection Reagent (GE Healthcare) with an ImageQuant LAS 4000 (GE Healthcare). To detect different proteins in the same membrane, conjugated antibodies were removed using stripping solution (62.5 mM Tris–HCl, pH = 6.8, 2% SDS, and 0.7% β-mercaptoethanol) by gently shaking for 50 min at 50 °C. After washing with PBST, blocking and antibody reactions were carried out.

### Intracellular ROS and mitochondrial analyses

Collected BM cells were stained with antibodies (biotinylated antibodies for lineage cells, streptavidin-APC780, c-Kit-APC, and Sca1-PECy7). After the antibody staining, the cells were mixed and reacted with Mitotracker Green, tetramethylrhodamine (TMRM; Thermo Fisher Scientific), Mitosox Red (Thermo Fisher Scientific), or CM-H2CDCFDA (2′,7′-dichlorodihydrofluorescein diacetate; Thermo Fisher Scientific). Cells were then washed and analysed by the FACS Canto II or FACsLyric.

### Quantitative PCR

LSK cells were sorted by fluorescence-activated cell sorting and collected by centrifugation. RNA was extracted with a RNeasy Mini Kit (Qiagen) and cDNA was synthesised with a QuantiTect Reverse Transcription Kit (Qiagen). The cDNA was used for real time PCR with TB green (Takara). PCR primers were as follows:

*Nqo1*^12^, 5′-AGTACAATCAGGGCTCTTCTCG-3′, 5′-AGCGTTCGGTATTACGATCC-3′; *Gstm1*^12^, 5′-CTACCTTGCCCGAAAGCAC-3′, 5′-ATGTCTGCACGGATCCTCTC-3′; *Actb*, 5′-CCTCTATGCCAACACAGTGC-3′, 5′-CCTGCTTGCTGATCCACATC-3′.

### Microarray and gene set analyses

BM cells of *Atg5*^*f*/*f*^;*Vav* and *Atg5*^*w*/*w*^;*Vav* mice were collected and LSK cells were stained as described above. LSK cells (> 1 × 10^4^ cells) were sorted directly into TRIzol (Invitrogen). Subsequent sample preparation and analysis were carried out by Hokkaido System Science. Details are shown in a previous report^62^. Normalised expression data were examined by Gene Set Enrichment Analysis (GSEA) v4.0.1 software (Broad Institute) using the GO gene set. The number of permutations was set to 1000.

### Statistical analysis

Unless indicated otherwise, data are shown as the mean ± SD. Dots on bar graphs indicate values of individual mice. Statistical differences between groups were determined by the Student’s unpaired t-test using Prism 5 software (GraphPad). *P < 0.05, **P < 0.01, ***P < 0.001, and ****P < 0.0001. In survival assays, statistical differences between groups were determined by the Mantel-Cox test.

### Data availability

The accession number for the microarray data in this study is GEO: GSE153613.

## Supplementary information

Supplementary Information.
