# Supplementary material for: Essential role of autophagy in protecting neonatal haematopoietic stem cells from oxidative stress in a p62-independent manner
Source: Sci Rep. 2021 Jan 18;11:1666. doi: 10.1038/s41598-021-81076-z (PMC7814027; doi:10.1038/s41598-021-81076-z)
Supplement: Supplementary file 1 — Supplementary Information. [file 41598_2021_81076_MOESM1_ESM.pdf]

Essential role of autophagy in protecting neonatal haematopoietic stem cells from oxidative stress in a p62-independent manner

Naho Nomura[1,7], Chiaki Ito[1,7], Takako Ooshio[1], Yuko Tadokoro[1,2], Susumu Kohno[3], Masaya Ueno[1,2], Masahiko Kobayashi[1,2], Atsuko Kasahara[4], Yusuke Takase[1], Kenta Kurayoshi[1], Sha Si[1,2], Chiaki Takahashi[3], Masaaki Komatsu[5], Toru Yanagawa[6], Atsushi Hirao[1,2*]

[1]Division of Molecular Genetics, Cancer and Stem Cell Research Program, Cancer Research Institute, Kanazawa University, Kakuma-machi, Kanazawa, Ishikawa 920-1192, Japan
[2]WPI Nano Life Science Institute (WPI-Nano LSI), Kanazawa University, Kakuma-machi, Kanazawa, Ishikawa 920-1192, Japan
[3]Division of Oncology and Molecular Biology, Cancer and Stem Cell Research Program, Cancer Research Institute, Kanazawa University, Kakuma-machi, Kanazawa, Ishikawa 920-1192, Japan
[4]Institute for Frontier Science Initiative, Kanazawa University, Kakuma-machi, Kanazawa, Ishikawa 920-1192, Japan
[5]Department of Physiology, Juntendo University Graduate School of Medicine, 2-1-1, Hongo, Bunkyo-ku, Tokyo 113-8421, Japan
[6]Faculty of Medicine, University of Tsukuba, 1-1-1 Tennodai, Tsukuba, Ibaraki 305-8575, Japan
[7]These authors contributed equally to this work.
*Corresponding author.

List of Supplementary Information

1. **Supplementary Fig.S1-S12**
2. **Supplementary Methods**

Supplementary Fig. S1

a

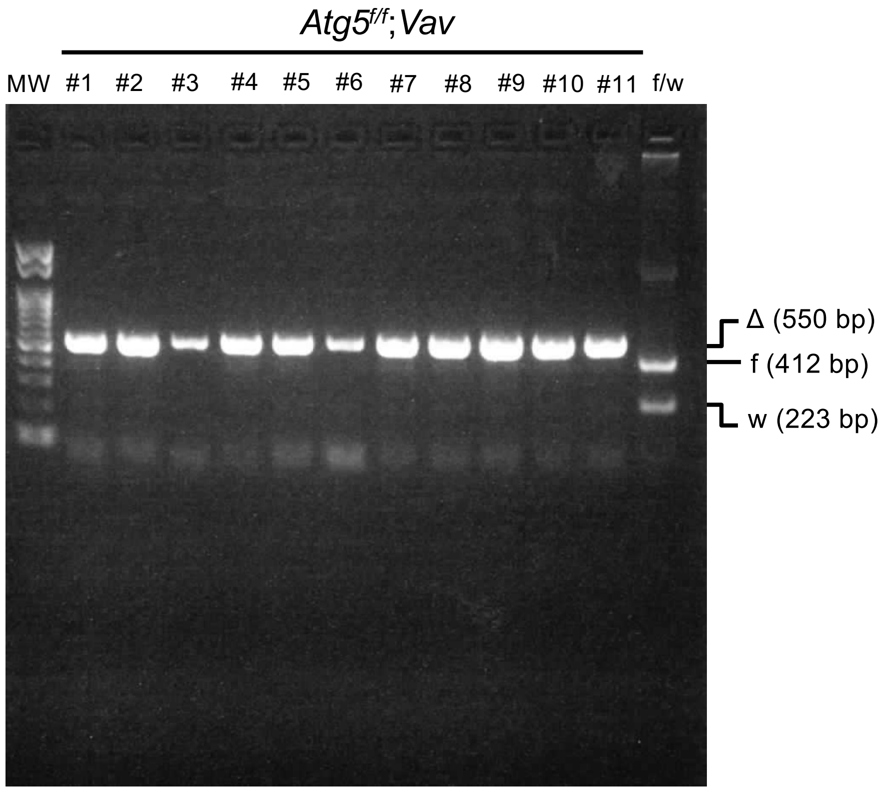

b

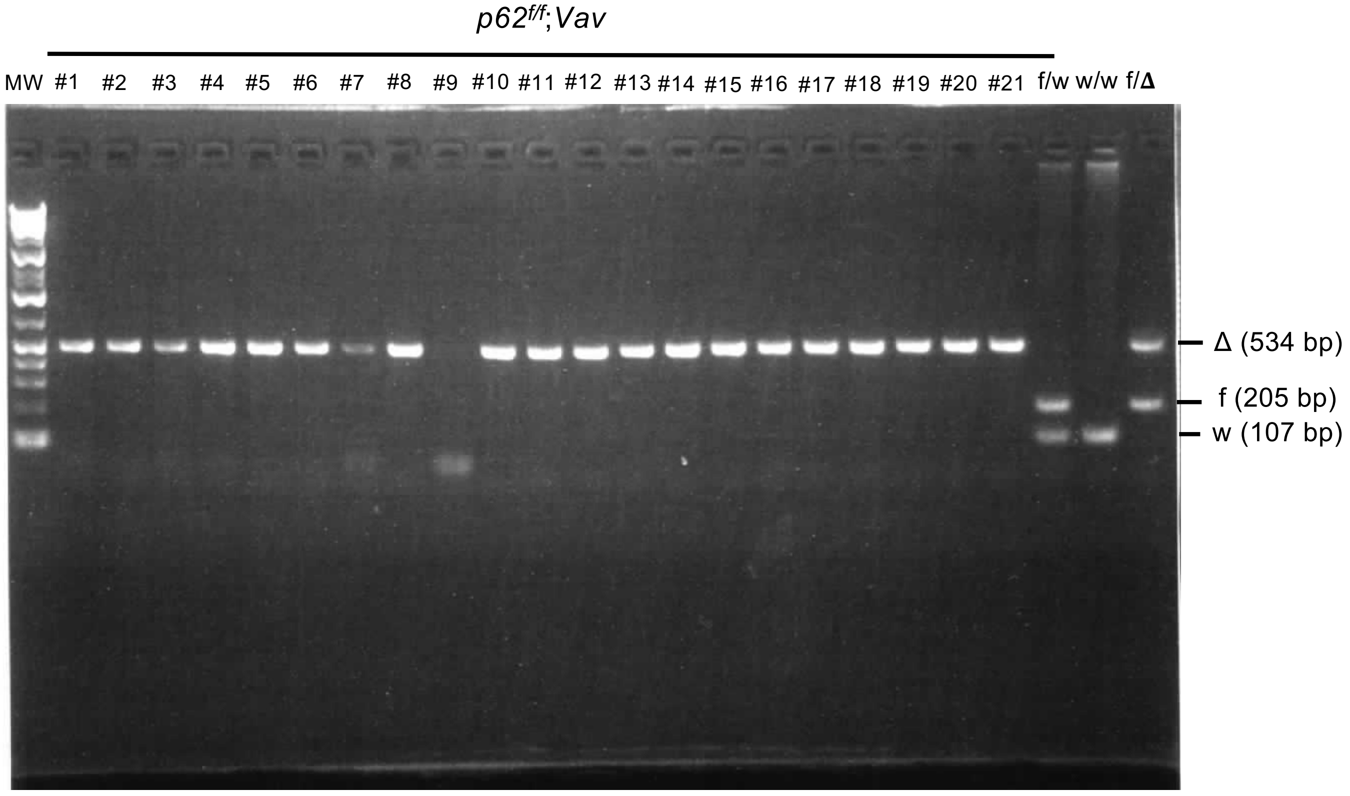

Colony PCR to determine *Atg5* and *p62* gene deletion efficiencies in LSK cells from *Atg5*$^{f/f}$;*Vav* and *p62*$^{f/f}$;*Vav* mice. Electrophoresis of PCR products derived from each colony (#1–21) are shown. (a) Colony PCR to determine *Atg5* gene deletion efficiencies in LSK cells from *Atg5*$^{f/f}$;*Vav* mice. (b) Colony PCR to determine *p62* gene deletion efficiencies in LSK cells from *p62*$^{f/f}$;*Vav* mice. For a colony, #9, DNA extraction failed.

Supplementary Fig. S2

a

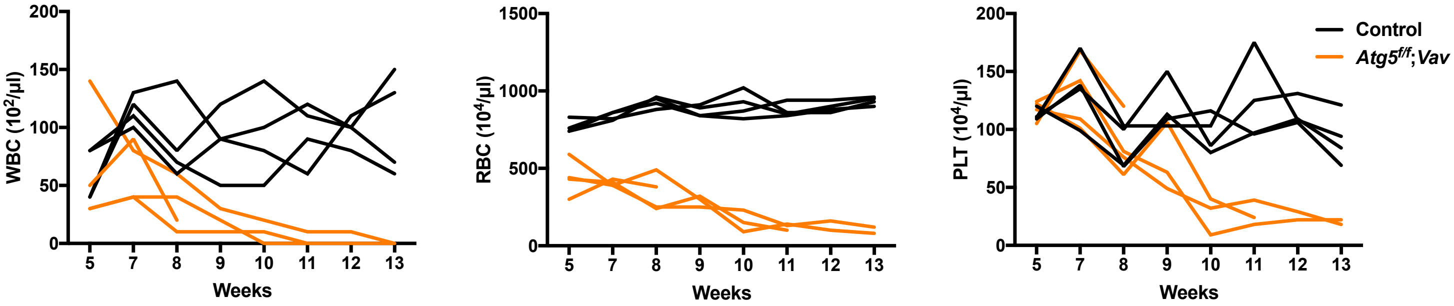

b

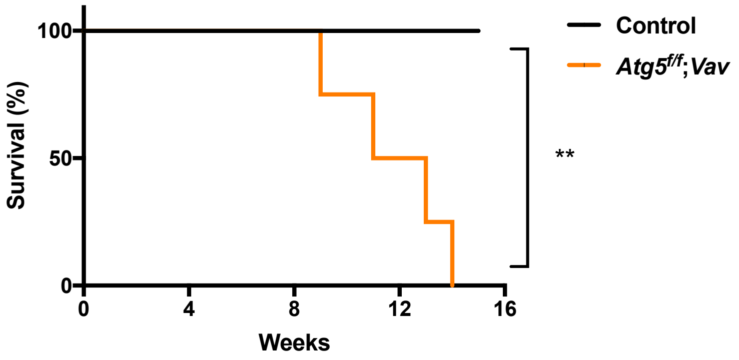

Peripheral blood cell counts in control and *Atg5$^{f/f}$;Vav* mice and their survival. (a) Counts of white blood cells (WBCs), red blood cells (RBCs), and platelets (PLTs) in PB of control (n=4) and *Atg5$^{f/f}$;Vav* (n=4) mice at the indicated weeks after birth. (b) Survival curve of control (n=4) and *Atg5$^{f/f}$;Vav* (n=4) mice.

Supplementary Fig. S3

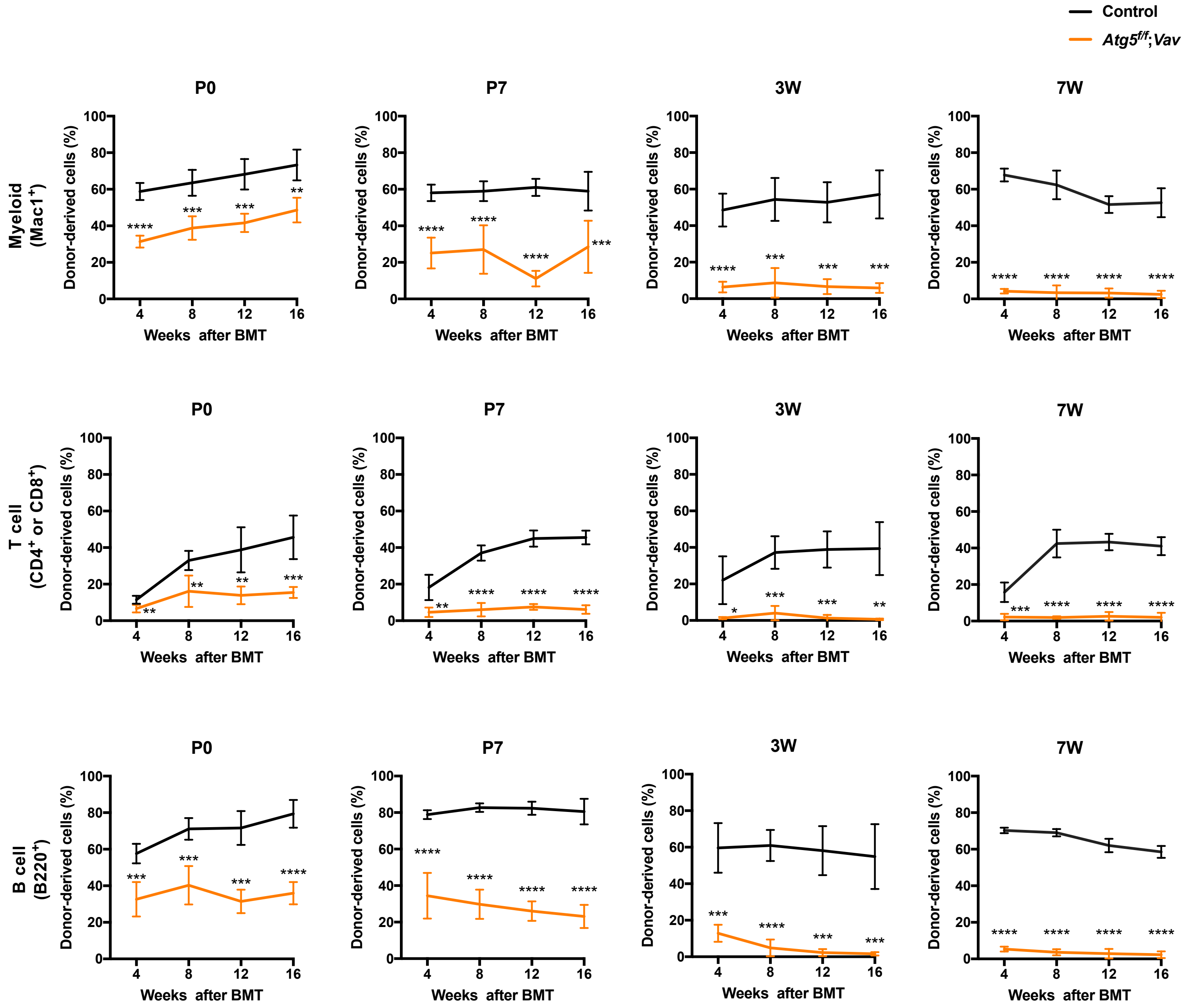

Competitive reconstitution analysis of BM cells from control (*Atg5*$^{f/w}$;*Vav)* and *Atg5*$^{f/f}$;*Vav* at P0, P7, 3 weeks, and 7 weeks. Frequencies of donor-derived cells among myeloid cells (Mac1^{+}; top), T cells (CD4^{+} or CD8^{+}; middle), and B cells (B220^{+}; top) in PB are shown. Data are the mean ± SD (n=4–5).

Supplementary Fig. S4

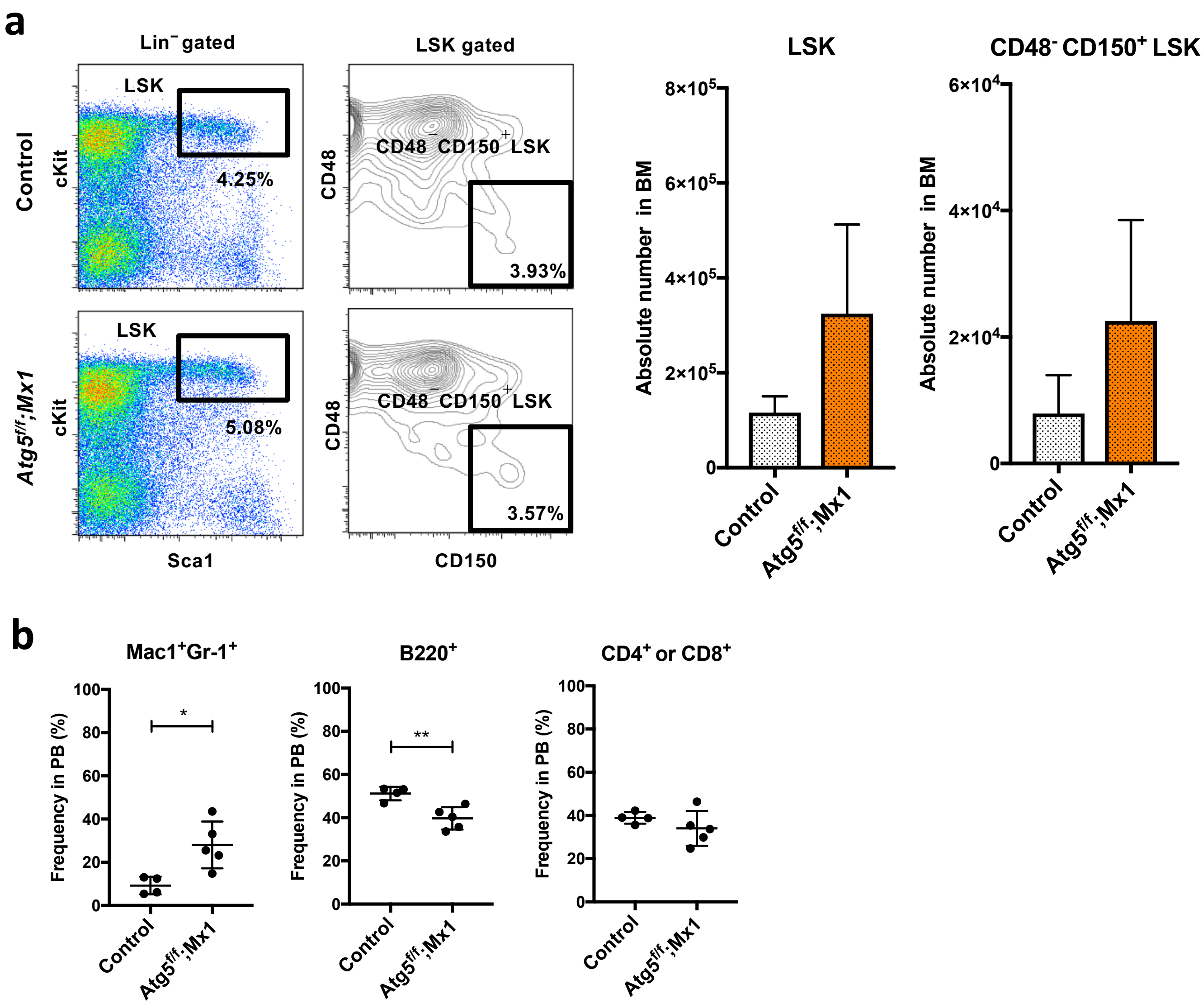

Comparative analysis of HSCs in BM and lineage cells in PB from control and *Atg5*$^{f/f}$;*Mx1* mice. (a) HSC population in control (*Atg5*$^{f/w}$;*Mx1*) and *Atg5*$^{f/f}$;*Mx1* mice at 4 weeks after p(I:C) administration. Left panel shows a representative flow cytometric plot of LSK (Lin⁻ c-Kit⁺ Sca1⁺) cells and CD48⁻CD150⁺LSK cells in at least three independent experiments. Right panel shows absolute numbers of LSK and CD48⁻CD150⁺LSK cells. Data are the mean ± SD (n=3). (b) Frequencies of myeloid cells (Mac1⁺Gr-1⁺), B cells (B220⁺), and T cells (CD4⁺ or CD8⁺) in PB. Data are the mean ± SD (n=4–5). Horizontal line indicates the mean of values.

Supplementary Fig. S5

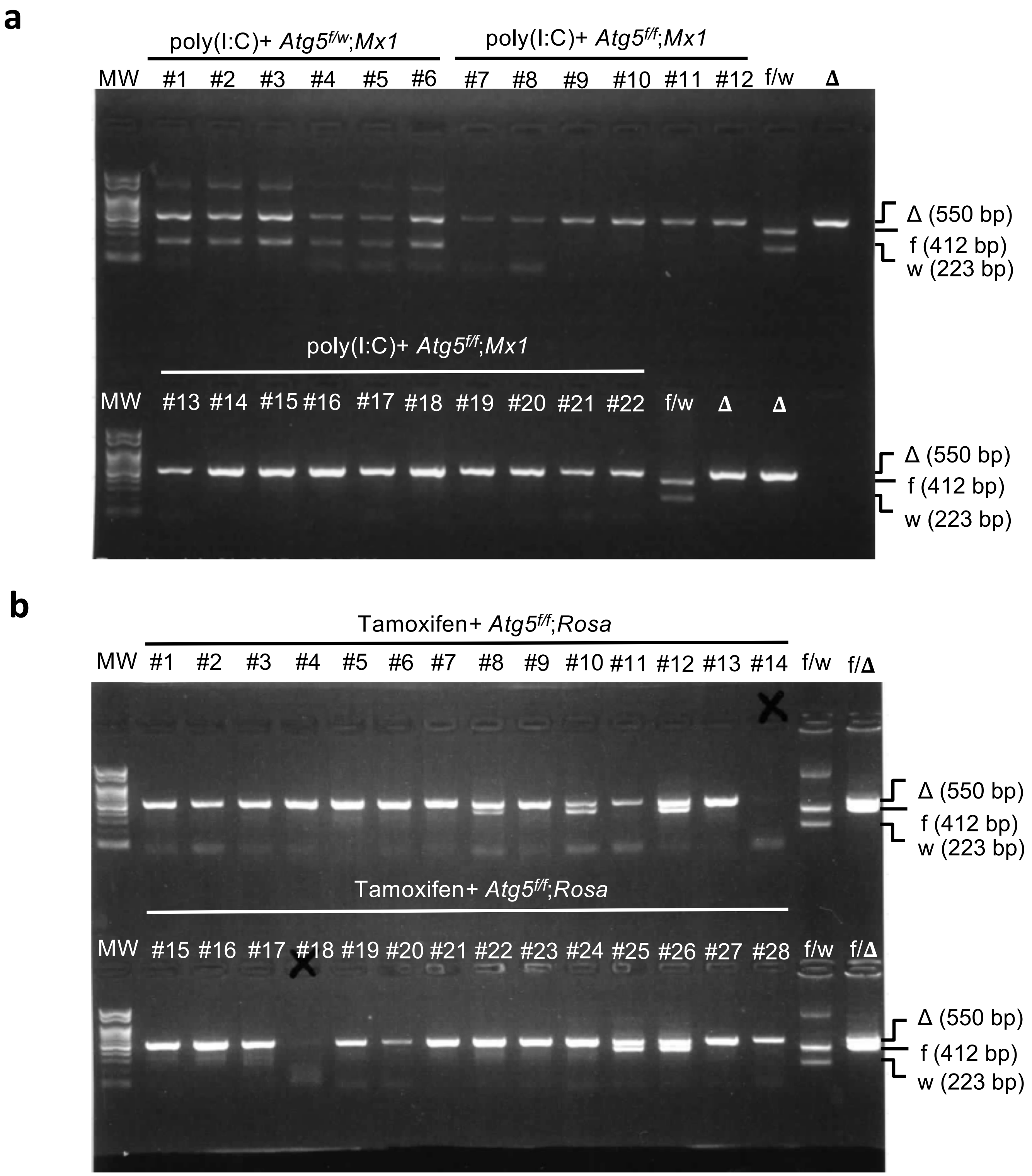

Colony PCR to determine *Atg5* gene deletion efficiencies in LSK cells from poly (I:C)-administrated *Atg5*$^{f/f}$;*Mx1* mice and tamoxifen-administrated *Atg5*$^{f/f}$;*Rosa* mice. Electrophoresis of PCR products derived from each colony (#1–28) are shown. (a) Detection of *Atg5* gene deletion in LSK cells from a *Atg5*$^{f/f}$;*Mx1* mouse and a *Atg5*$^{f/w}$;*Mx1* mouse. (b) Detection of *Atg5* gene deletion in LSK cells from a *Atg5*$^{f/f}$;*Rosa* mouse. For colonies, #14 and #18, DNA extraction failed.

Supplementary Fig. S6

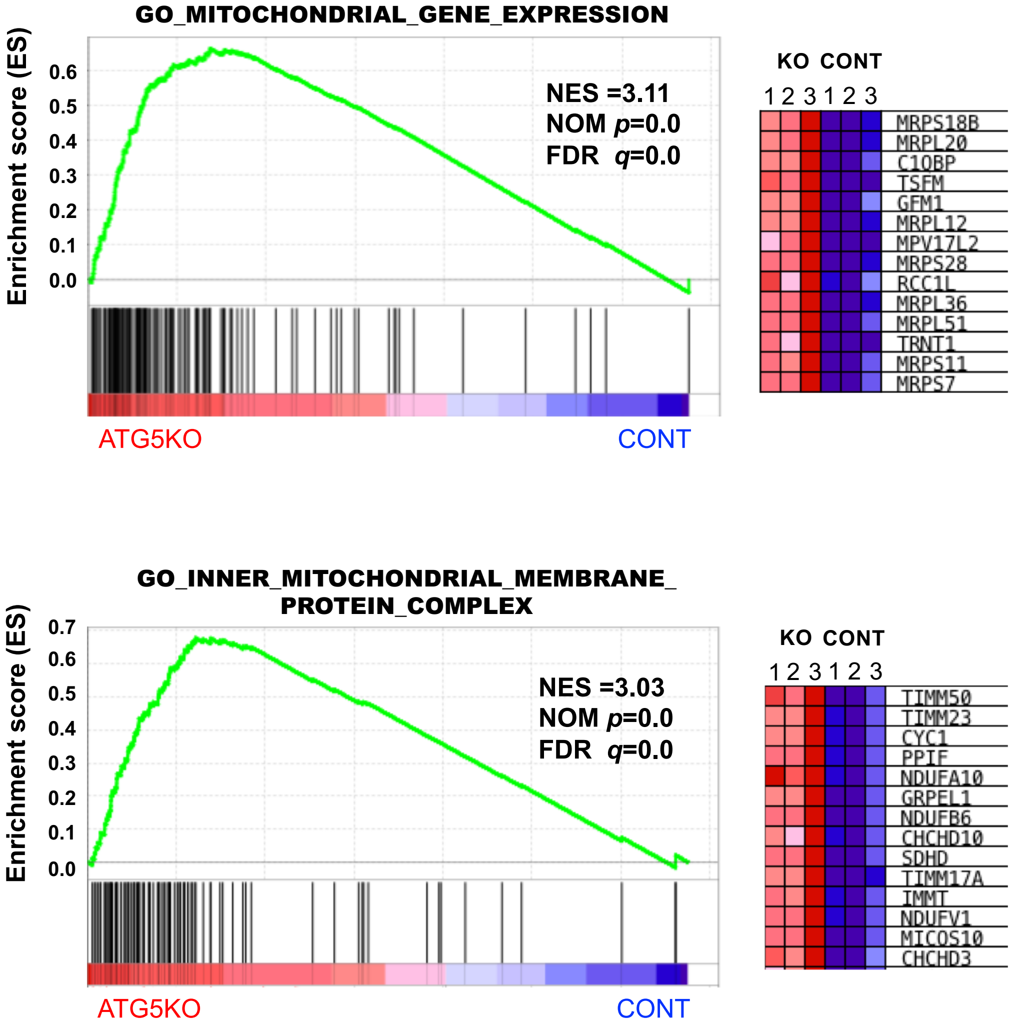

GSEA enrichment score curves of control (*Atg5*$^{w/w}$;*Vav*) and *Atg5*$^{f/f}$;*Vav* LSK cells analysed for gene sets associated with mitochondrial gene expression and the inner mitochondrial membrane protein complex. Heatmaps of representative gene expression in each gene set are shown at the right. NES, normalised enrichment score; NOM, nominal *p*-value; FDR, false discovery rate.

Supplementary Fig. S7

a

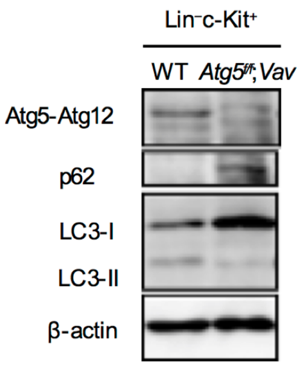

b

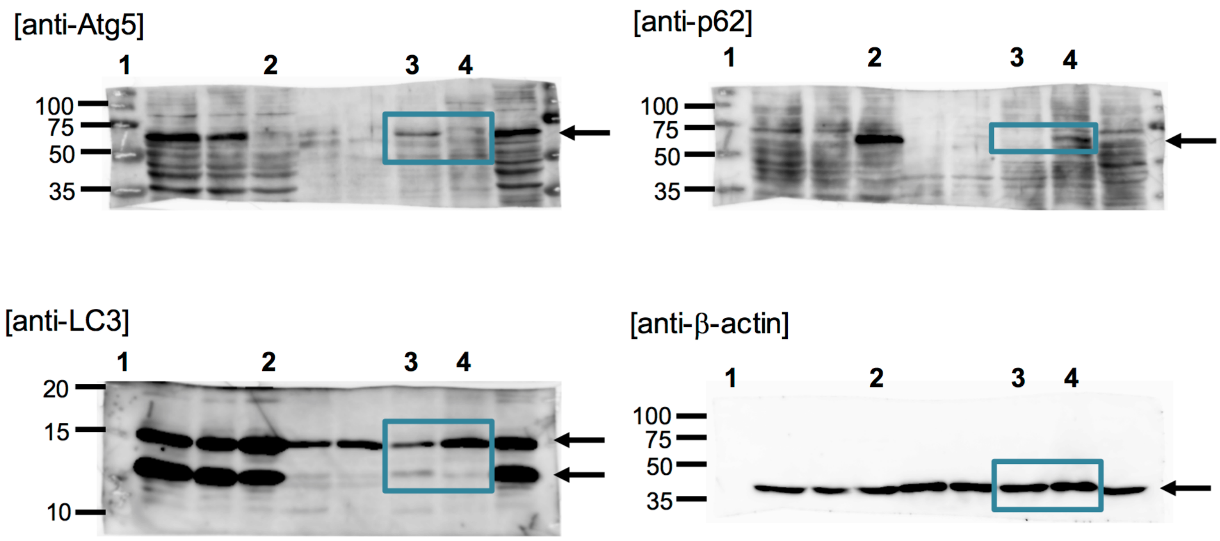

1: Molecular weight marker 2: Atg5-negative;p62-positive control
3: WT LK cells 4: *Atg5*$^{f/f}$;*Vav* LK cells

Western blot to detect the indicated proteins in LK (Lin$^-$c-Kit$^+$) cells from wildtype and *Atg5*$^{f/f}$;*Vav* mice at 3 weeks. (a) Cropped blots. (b) Uncropped images. Boxes highlight the cropped bands shown in (a). Transferred membranes were divided into upper and lower parts. The upper part was first reacted with an anti-Atg5 antibody and a Atg5 signal was detected. Then, the membrane was stripped and reacted with an anti-p62 antibody. After detection of p62 signals, the membrane was re-stripped and reacted with an anti-β-actin antibody. The lower part was reacted with an anti-LC3 antibody. Lane 1, molecular weight marker; Lane 2, Atg5-negative and p62-positive control; Lane 3, LK (Lin$^-$c-Kit$^+$) cells from wildtype mice; Lane 4, LK (Lin$^-$c-Kit$^+$) cells from *Atg5*$^{f/f}$;*Vav* mice. Other irrelevant samples were loaded in unlabelled lanes. Large blank spaces and other parts of divided membranes in the full size image were cropped for presentation.

Supplementary Fig. S8

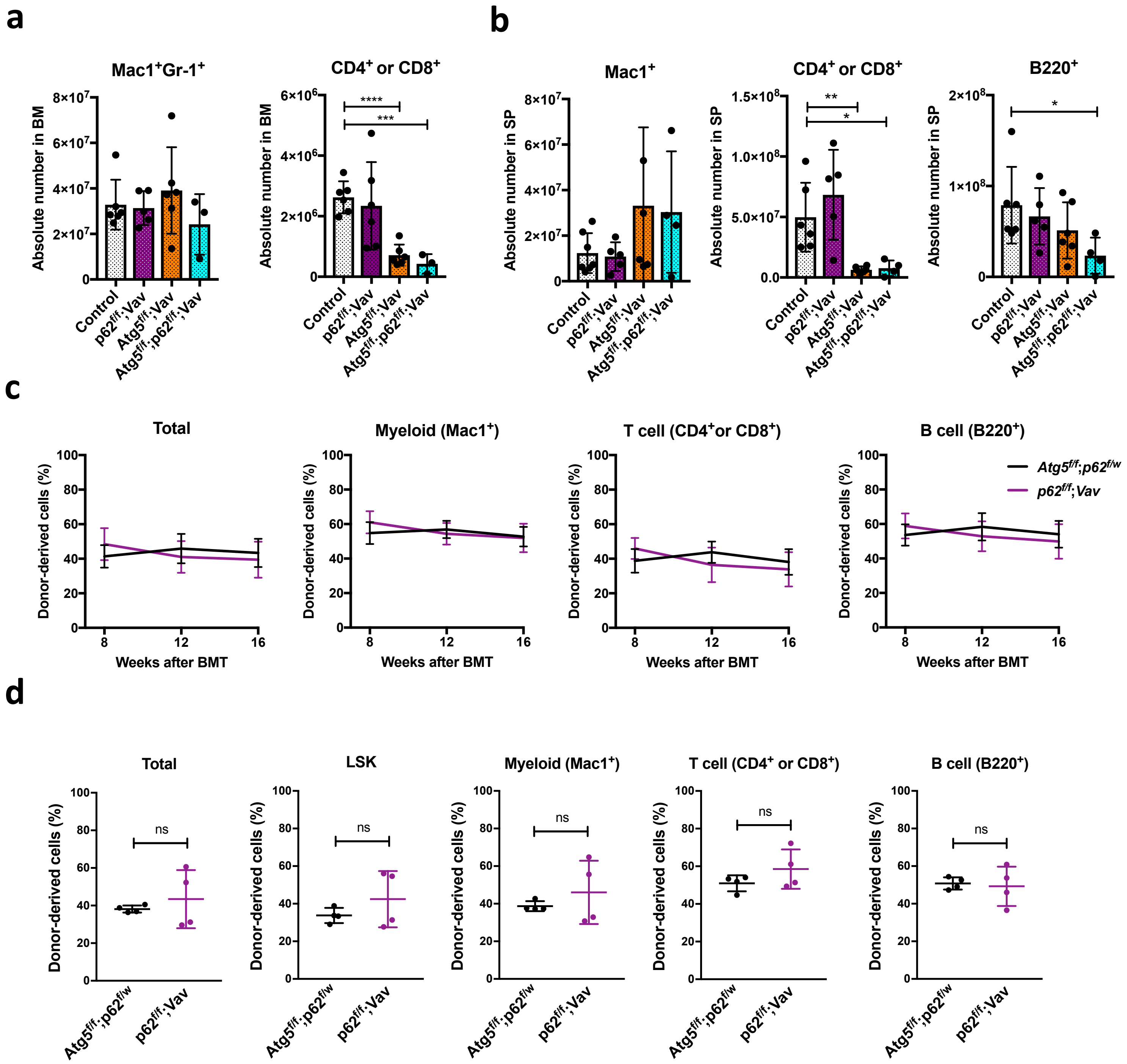

Comparative analysis of lineage cells in BM and spleen of control, *p62*$^{f/f}$;*Vav*, *Atg5*$^{f/f}$;*Vav*, and *Atg5*$^{f/f}$;*p62*$^{f/f}$;*Vav* mice, and competitive reconstitution analysis of BM cells from control and *p62*$^{f/f}$;*Vav* mice. (a) Absolute numbers of myeloid cells (Mac1$^+$Gr-1$^+$) and T cells (CD4$^+$ or CD8$^+$) in BM at 7 weeks. Data are the mean ± SD (n=3–6). Each dot indicates the value of individual mice. (b) Absolute numbers of myeloid cells (Mac1$^+$), T cells (CD4$^+$ or CD8$^+$), and B cells (B220$^+$) in spleen. Data are the mean ± SD (n=4–6). Each dot indicates the value of individual mice. (c) Competitive reconstitution analysis of BM cells from *Atg5*$^{f/f}$;*p62*$^{f/w}$ and *p62*$^{f/f}$;*Vav* mice at 7 weeks. Frequencies of donor-derived cells among total cells and lineage cells in PB were analysed 8–16 weeks after transplantation. Data are the mean ± SD (n=4). (d) Frequencies of donor-derived cells among total, LSK cells, myeloid cells (M), T cells (T), and B cells (B) in BM were analysed at 16 weeks after transplantation. Data are the mean ± SD (n=4). Horizontal line indicates the mean of values.

Supplementary Fig. S9

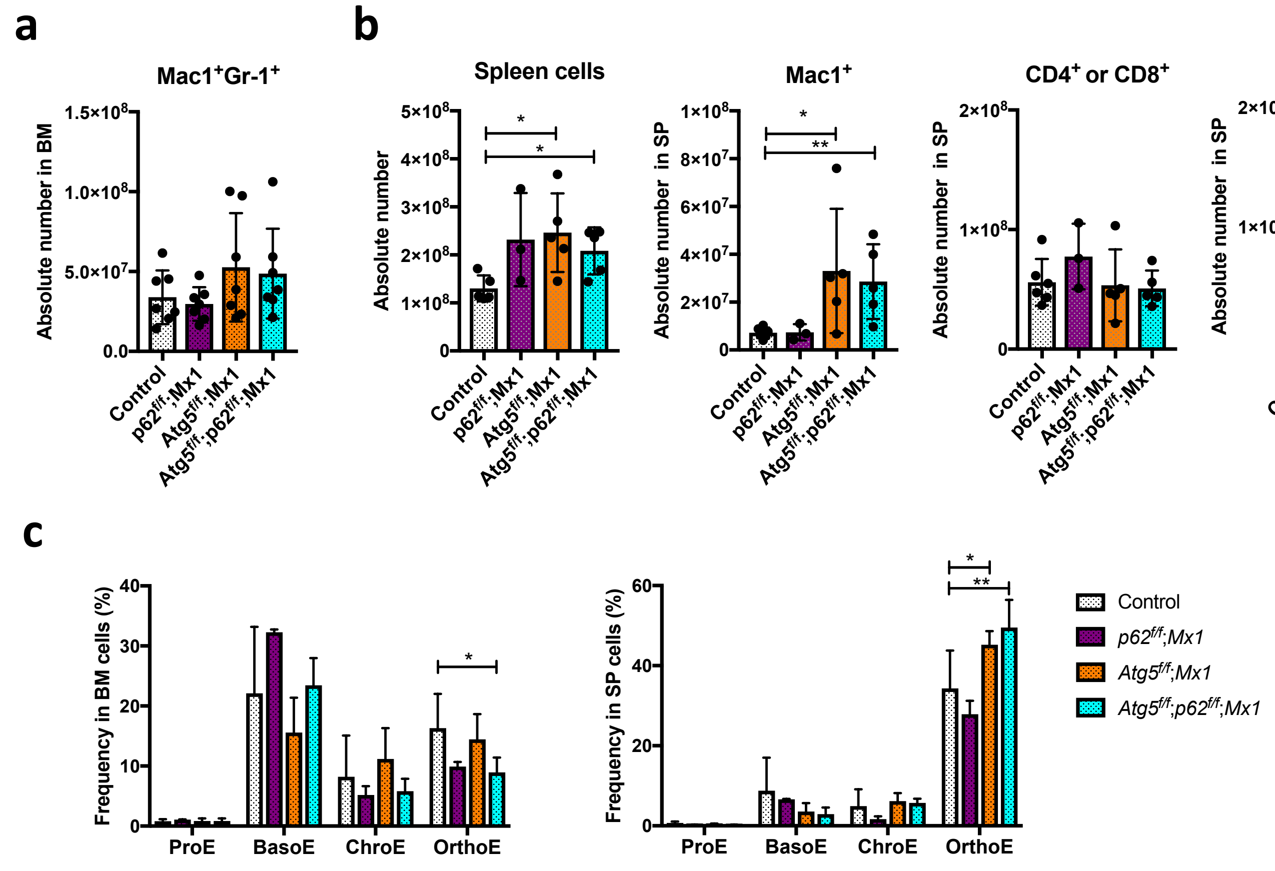

Comparative analysis of lineage cells in BM and spleen of control, *p62^f/f^;Mx1*, *Atg5^f/f^;Mx1*, *Atg5^f/f^;p62^f/f^;Mx1* mice. (a) Absolute numbers of myeloid cells (Mac1$^+$Gr-1$^+$) in BM. Data are the mean ± SD (n=7). (b) Absolute numbers of total spleen cells, myeloid (Mac1$^+$) cells, T cells (CD4$^+$or CD8$^+$), and B cells (B220$^+$) in spleen. Data are the mean ± SD (n=3–6). (c) Erythroid development in BM and spleen. Frequencies of ProE (Ter119^{low}CD71^{high}), BasoE (Ter119^{high}CD71^{high}), ChroE (Ter119^{high}CD71^{int}), and OrthoE (Ter119^{high}CD71^{low}) cells among BM cells (left) and spleen cells (right) are shown. Data are mean ± SD (n=3–7).

Supplementary Fig. S10

a

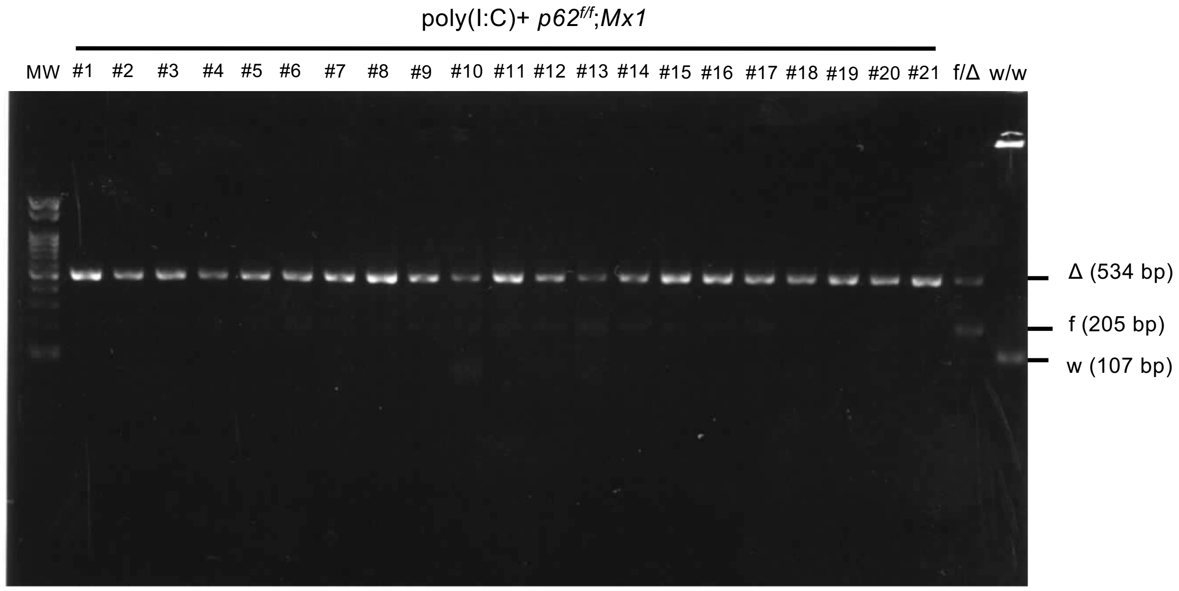

b

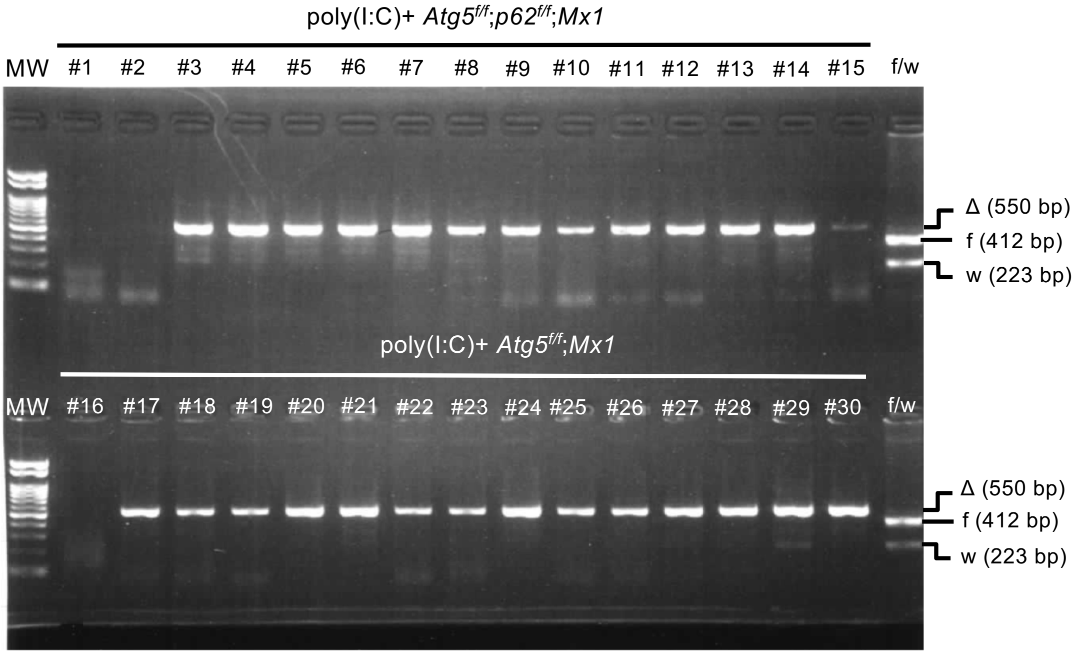

c

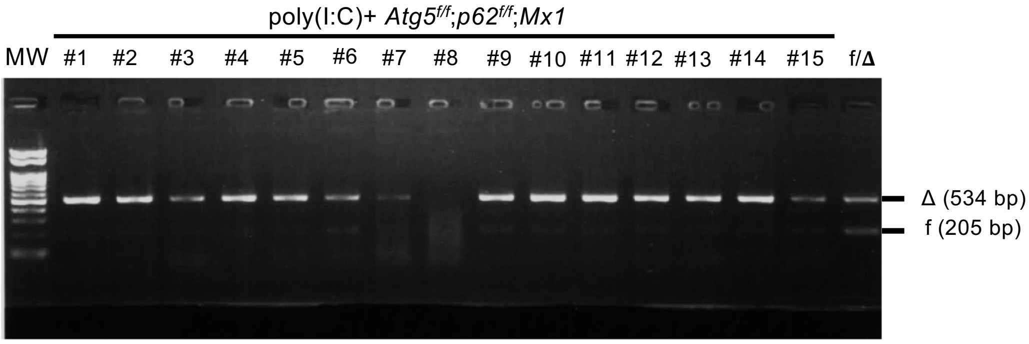

Colony PCR to determine *p62* and *Atg5* gene deletion efficiencies in LSK cells from poly (I:C)-administrated *p62*$^{f/f}$;*Mx1* and *Atg5*$^{f/f}$;*p62*$^{f/f}$;*Mx1* mice. Electrophoresis of PCR products derived each colony (#1–30) are shown. (a) Detection of *p62* deletion in LSK cells from a *p62*$^{f/f}$;*Mx1* mouse. (b) Detection of *Atg5* deletion in LSK cells from a *Atg5*$^{f/f}$;*p62*$^{f/f}$;*Mx1* mouse and *Atg5*$^{f/f}$;*Mx1* mouse. For colonies, #1, #2, and #16, DNA extraction failed. (c) Detection of *p62* deletion in LSK cells from a *Atg5*$^{f/f}$;*p62*$^{f/f}$;*Mx1* mouse. For a colony, #8, DNA extraction failed.

Supplementary Fig. S11

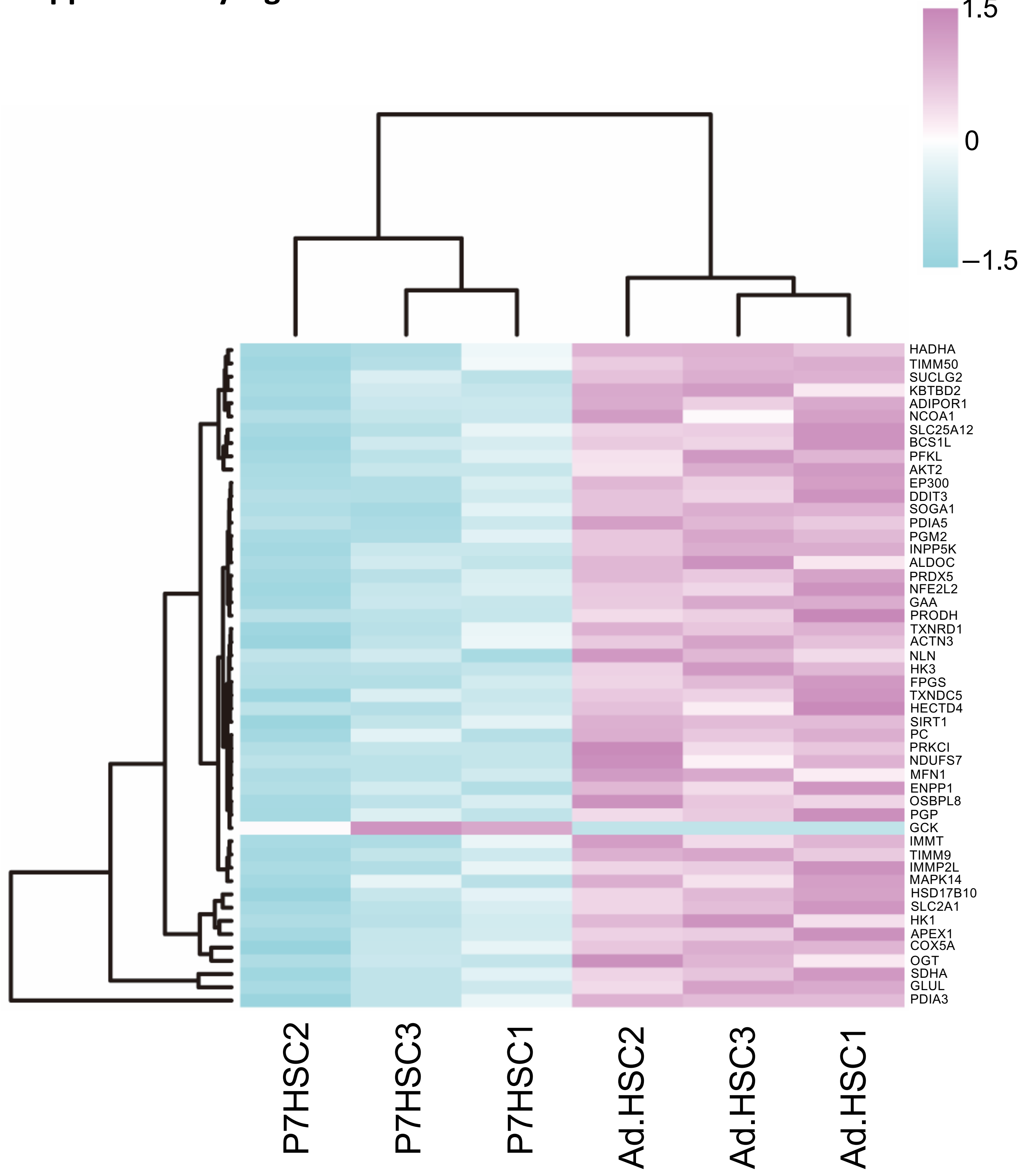

Heatmap of comparative genes expression differences between neonatal (P7) and adult HSCs. A heatmap of the top 50 differentially expressed genes involved in glucose metabolism, glutamine metabolism, mitochondria, and redox regulation is shown. Samples and genes were clustered in accordance with gene expression (mean centered and scaled normalized counts).Public RNA-seq data of GSE128762 (GSM3684505, GSM3684506, GSM3684507, GSM3684518, GSM3684519, and GSM3684520) were used for the analysis[40].

Supplementary Fig. S12

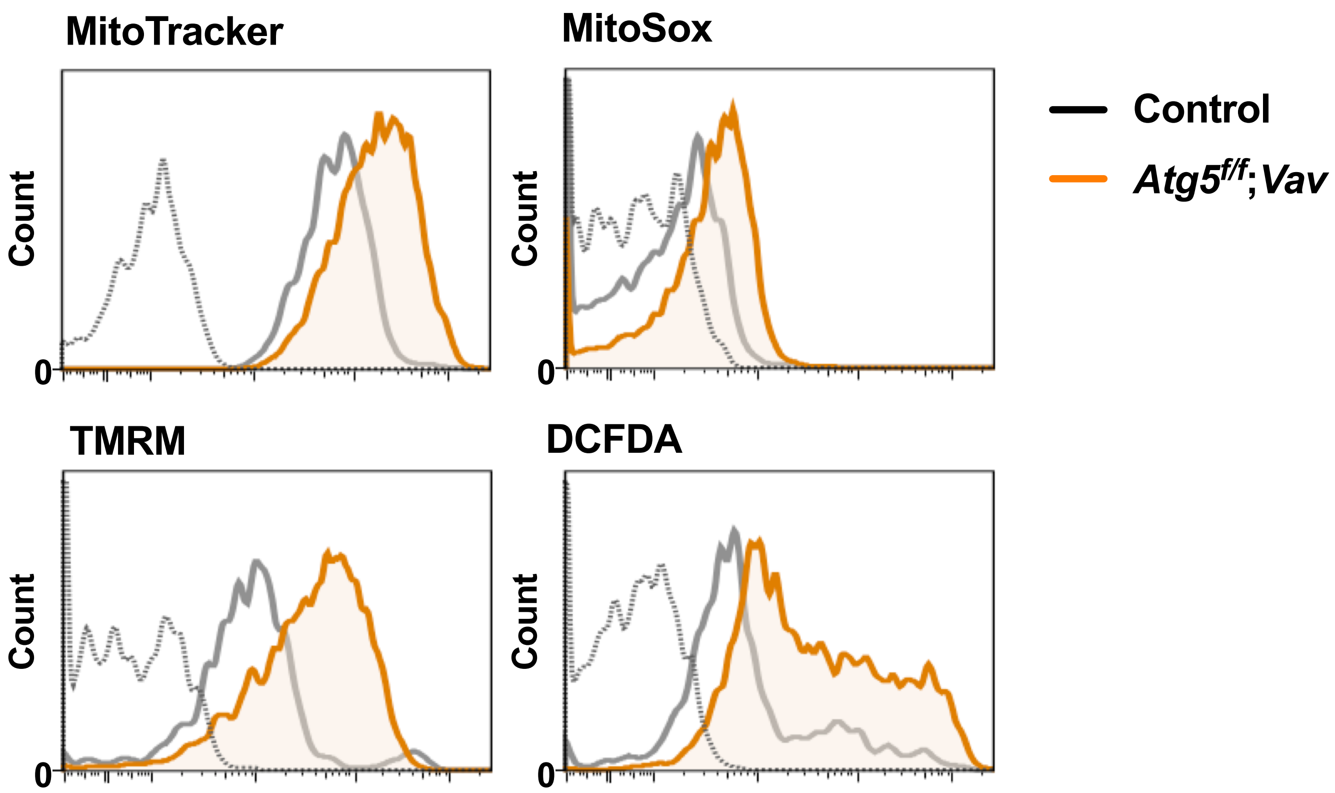

Flow cytometric analysis of MitoTracker green, MitoSox Red, TMRM, and DCFDA staining in LSK cells from control and *Atg5*$^{f/f}$;*Vav* mice at 7 weeks. Representative data from two independent experiments are shown.

Supplementary Methods

RNA-seq analysis

Single ended raw sequencing reads were extracted using fasterq and mapped with Kallisto index built from the human cDNA (hg38) downloaded from Ensemble server. The transcript abundance from Kallisto was normalized as a transcript per million (TPM) using tximport of R package. Gene list involved in glucose metabolism, glutamine metabolism, mitochondria, and redox regulation were built from gene set of MSigDB (M13870, M22685, M10955, M17748, M12813). Differential gene expression was carried out using edgeR package.
